# Human coronary microvascular contractile dysfunction associates with viable synthetic smooth muscle cells

**DOI:** 10.1093/cvr/cvab218

**Published:** 2021-06-26

**Authors:** Kim A Dora, Lyudmyla Borysova, Xi Ye, Chloe Powell, Timea Z Beleznai, Christopher P Stanley, Vito D Bruno, Tobias Starborg, Errin Johnson, Anna Pielach, Michael Taggart, Nicola Smart, Raimondo Ascione

**Affiliations:** The Vascular Pharmacology Group, Department of Pharmacology, University of Oxford, Mansfield Road, Oxford, OX1 3QT, UK; The Vascular Pharmacology Group, Department of Pharmacology, University of Oxford, Mansfield Road, Oxford, OX1 3QT, UK; The Vascular Pharmacology Group, Department of Pharmacology, University of Oxford, Mansfield Road, Oxford, OX1 3QT, UK; The Vascular Pharmacology Group, Department of Pharmacology, University of Oxford, Mansfield Road, Oxford, OX1 3QT, UK; The Vascular Pharmacology Group, Department of Pharmacology, University of Oxford, Mansfield Road, Oxford, OX1 3QT, UK; The Vascular Pharmacology Group, Department of Pharmacology, University of Oxford, Mansfield Road, Oxford, OX1 3QT, UK; Bristol Heart Institute and Translational Biomedical Research Centre, University of Bristol, Bristol Royal Infirmary, Upper Maudlin Street, Bristol, BS2 8HW, UK; Division of Cell Matrix Biology and Regenerative Medicine School of Biological Sciences Faculty of Biology, Medical and Health Sciences, University of Manchester, B.3016 Michael Smith Building, Oxford Road, Manchester, M13 9PT, UK; Sir William Dunn School of Pathology, University of Oxford, South Parks Road, Oxford, OX1 3RE, UK; Sir William Dunn School of Pathology, University of Oxford, South Parks Road, Oxford, OX1 3RE, UK; Biosciences Institute, Newcastle University, International Centre for Life, Central Parkway, Newcastle upon Tyne, NE1 3BZ, UK; Department of Physiology, Anatomy and Genetics, University of Oxford, Sherrington Building, Parks Road, Oxford, OX1 3PT, UK; Bristol Heart Institute and Translational Biomedical Research Centre, University of Bristol, Bristol Royal Infirmary, Upper Maudlin Street, Bristol, BS2 8HW, UK

**Keywords:** Human, Coronary arterioles, Coronary microvascular function, Myogenic tone, Ultrastructure, Ca2+, signalling, Synthetic phenotype, Smooth muscle cell, Heart valve disease, Microvascular perfusion

## Abstract

**Aims:**

Coronary microvascular smooth muscle cells (SMCs) respond to luminal pressure by developing myogenic tone (MT), a process integral to the regulation of microvascular perfusion. The cellular mechanisms underlying poor myogenic reactivity in patients with heart valve disease are unknown and form the focus of this study.

**Methods and results:**

Intramyocardial coronary micro-arteries (IMCAs) isolated from human and pig right atrial (RA) appendage and left ventricular (LV) biopsies were studied using pressure myography combined with confocal microscopy. All RA- and LV-IMCAs from organ donors and pigs developed *circa* 25% MT. In contrast, 44% of human RA-IMCAs from 88 patients with heart valve disease had poor (<10%) MT yet retained cell viability and an ability to raise cytoplasmic Ca^2+^ in response to vasoconstrictor agents. Comparing across human heart chambers and species, we found that based on patient medical history and six tests, the strongest predictor of poor MT in IMCAs was increased expression of the synthetic marker caldesmon relative to the contractile marker SM-myosin heavy chain. In addition, high resolution imaging revealed a distinct layer of longitudinally aligned SMCs between ECs and radial SMCs, and we show poor MT was associated with disruptions in these cellular alignments.

**Conclusion:**

These data demonstrate the first use of atrial and ventricular biopsies from patients and pigs to reveal that impaired coronary MT reflects a switch of viable SMCs towards a synthetic phenotype, rather than a loss of SMC viability. These arteries represent a model for further studies of coronary microvascular contractile dysfunction.

## Graphical Abstract

**Graphical Abstract:**
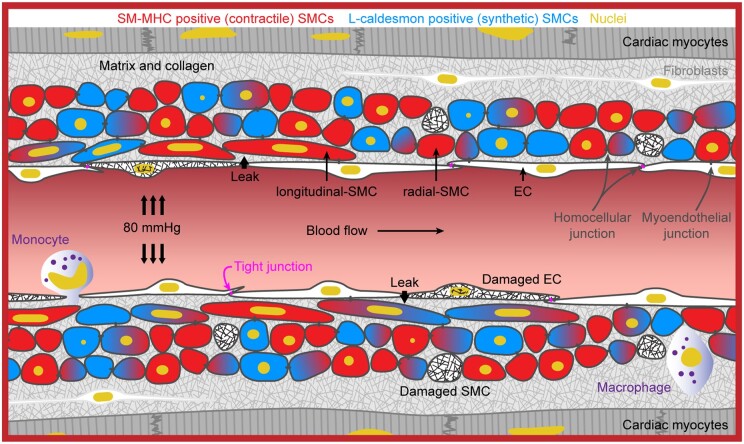
A synthetic SMC phenotype underlies coronary microvascular contractile dysfunction. The profile of biomarkers for contractile vs. synthetic phenotype strongly reflects whether an artery can generate MT and therefore contribute to the regulation of coronary blood flow.

## 1. Introduction

Coronary vascular smooth muscle cells (SMCs) exert physiological functions through both contractile and synthetic SMC phenotypes, together maintaining effective function of the coronary circulation.^1,^^2^ Within the coronary microcirculation, contractile SMCs exert myogenic reactivity and tone,^3,^^4^ which is an important regulator of myocardial perfusion.^3–9^ Furthermore, contractile SMCs react to vasoactive factors acting directly on SMCs or via the endothelium, often released and acting in response to metabolic demand.^3–9^ In parallel, synthetic SMCs secrete extracellular matrix components and may increase proliferation and migration during vessel remodelling, e.g. exercise, pregnancy, or vascular repair.^1,^^2,^^10^ The role of contractile and synthetic SMCs in intramyocardial coronary micro-arteries (IMCAs) in patients with no significant coronary ischaemic disease has not been investigated. Coronary microvascular dysfunction in the absence of obstructive coronary disease is associated with angina-like chest pain, heart failure, inflammation, and poor prognosis.^11–17^ However, the causes of the microvascular dysfunction, in particular poor myogenic reactivity, in isolated human IMCAs (h-IMCAs) from viable myocardia are not known, and this knowledge is of prime importance for myocardial function.

Normal or dysfunctional IMCAs cannot be imaged directly *in vivo* due to the technological limitations of modern *in vivo* imaging,^16,^^18,^^19^ despite the clear need to do so for effective treatment strategies.^20^ Instead, indexes of microvascular dysfunction are based primarily on changes in response to vasodilators^17,^^18^ or on indirect imaging evaluations, such as myocardial perfusion scans based on myocardial uptake of contrast dye or radioisotopes.^21^ The more recent use of pressure and temperature wires to provide raw values for an index of microvascular resistance are clear advances,^22^ but do not separate myogenic tone (MT) from other influences, such as vascular remodelling and vasoconstrictors. Instead, h-IMCAs are studied *ex vivo*, isolated from human right atrial appendage biopsies (h-RA-IMCAs). These small coronary arteries and arterioles are myogenically active^23–25^ and respond to vasoconstrictors and both endothelium-dependent and -independent vasodilators.^23,^^24,^^26^ While their function has been studied for decades, little is known regarding their structure and how this influences function. Histological images of micro-arteries within fixed-tissue slices are available,^27^ and fluorescence imaging of h-IMCAs from right atrium have been reported,^6,^^24,^^28^ but these all lack paired assessment of MT and vascular reactivity. Perhaps most importantly, there are no *ex vivo* functional data from human left ventricular IMCAs (h-LV-IMCAs). At present no cardiac surgery allows sampling of the large biopsies (∼1 cm^2^) required for IMCA reactivity studies and sourcing organ donor biopsies for this purpose has not been achieved. The pig is arguably the most appropriate non-primate alternative^29^ with existing work based on sub-epicardial arterioles and large epicardial coronary arteries.^7,^^30–32^ However, no data are available using porcine atrial (p-RA-) or ventricular (p-LV-) IMCAs from fresh biopsies harvested at clinical standards.

The goal of this study was to use a sequence of conventional and newly established tests of SMC viability to identify pathways associated with poor MT of h-IMCAs from non-ischaemic myocardium.

## 2. Materials and methods

In brief, with fully expanded details available in the Supplementary material online.

### 2.1 Human study design

#### 2.1.1 Right atrial appendage surgical study participants

We enrolled 88 patients (aged ≥25 and ≤80 years) with no typical obstructive coronary disease and with valvular disease requiring either aortic valve replacement (AVR) or mitral valve repair/replacement (MVR) or both (AVR+MVR) undergoing elective or urgent cardiac surgery. Exclusion criteria are outlined in *Table 1*.

**Table 1:** Patient characteristics and exclusion criteria for h-RA-IMCAs (*n* = 88)

			*n*	%
	Sex (female/male)		29/59	100
	Age, year (mean±SD)	63±11	88	100
Cardiac disease	Valve disease		88	100
	Aortic		51	58
	Mitral		37	42
Risk factors	Treated hypertension		45	51
	Treated hypercholesterolaemia		31	35
	Diabetes mellitus		10	11
	Smoking history Current smokers		45 5	51 6
	Ex-smoker (>1 month)^a^		40	45
	None of the above		32	36
Baseline medications	Statins		32	36
	Diuretics		22	25
	Ca^2+^ channel blocker		12	14
	Beta-blocker		25	28
	Aspirin		20	23
	Anticoagulants		16	18
	ACE-inhibitors		29	33
Symptoms	NYHA heart failure class			
	I/II		12/43	63
	III		32	36
	IV		1	1
	CCS angina class			
	0		56	64
	I/II		4/19	26
	III/IV		6/3	10
Exclusion criteria	Age <25 years or >80 years			
	Severe coronary artery disease			
	Pulmonary hypertension >50 mmHg			
	Impaired right ventricular function (<35%)			
	Severely dilated atria (>5.0 cm)			
	Need for ascending/root aortic surgery			
	Emergency surgery			
	Acute endocarditis			
	Infection, known HIV, Hepatitis A, B, C			
	Cancer or receiving chemotherapy			
	Immune disease			
	Ongoing pregnancy			

SD, standard deviation; ACE, angiotensin converting enzyme; HIV, human immunodeficiency virus; NYHA, New York Heart Association; CCS, Canadian Cardiovascular Society.

a Ceased smoking at least 1 month before surgery.

##### 2.1.1.1 Consenting and confidentiality

Informed consent was obtained under and by the protocol No. 07Q160738 and No. 11SC0140. Any data information passed on to the Oxford Laboratory was anonymized. Any lab assay was carried out under blind conditions with respect to patient risk profile. The study fully complied with the Data Protection Act.

##### 2.1.1.2 Study settings

The study was set up in Oxford and extended to Bristol, where all the patients were recruited at the Bristol Heart Institute (BHI). The University Hospital Bristol NHS Foundation Trust sponsored the study in Bristol (Sponsor Lock code: 48676/124568/1/50). The trial was initially approved in 2012 by the Oxford Research Ethics Committee and extended to the Bristol site (Trust study No: CS/2012/4200; REC Reference No: 10/H0606/36) and amended in 2015. RA biopsy samples from recruited patients were transported to Oxford under a material transfer agreement under strict packaging, temperature regulation (∼10°C), and time-limit conditions. This research complies with the Helsinki Declaration.

##### 2.1.1.3 Surgical methods

**Figure 1:**
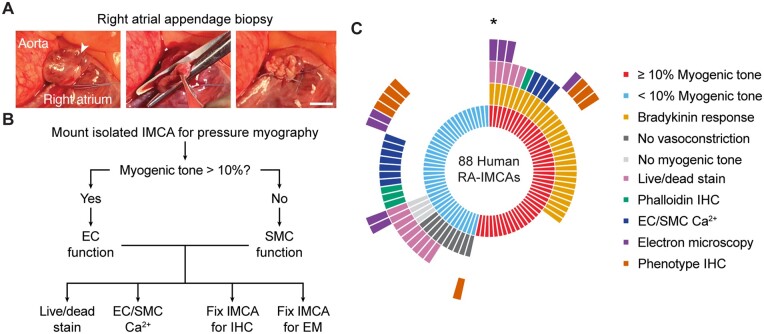
Experimental protocols. (*A*) For both human and porcine RA surgical biopsies a purse string suture was positioned around the tip of the appendage, the specimen excised, and the suture tied off and over sewn. Arrowhead, apex, bar =10 mm. (*B, C*) Sequence of experimental protocols in *ex vivo* cannulated arteries from patients with valve disease. Each radiating block in (*C*) represents one patient; e.g. the single micro-artery studied from the patient indicated by the asterisk developed ≥10% MT and dilated to BK, was imaged for live/dead cells, then fixed for EM, all while cannulated and pressurized to 80 mmHg.

Operations were carried out following standard protocols for the BHI. Baseline, surgical, and anaesthetic management were according to standardized protocols.^33^ Briefly, after pre-medication with temazepam, anaesthesia was induced with a combination of propofol and remifentanil; muscle relaxation was achieved using vecuronium. Anaesthesia was maintained by infusion of propofol and remifentanil (5 mg remifentanil to 1 g propofol). Right atrial (RA) biopsy (∼1 cm^2^) was always collected before starting cardiopulmonary bypass and prompt storage within 5–10 s in the preservation solution on ice and then couriered from Bristol to Oxford where they were assessed within 2–4 h following collection (*Figure 1*).

#### 2.1.2 Left ventricle biopsies from organ donors

Human heart tissue (Newcastle) was provided by The Newcastle Institute of Transplantation Tissue Biobank, 17/NE/0022, Research Ethics Committee North East-Newcastle and North Tyneside 1 Research Ethics Committee.

##### 2.1.2.1 Patient details

Females aged 56, 63, and 71 years. Left ventricular (LV) biopsies (>1cm^2^) were collected from the endocardial surface, and together with RA biopsies were immediately placed in preservation solution on ice, couriered from Newcastle to Oxford, and assessed within 5–8 h following collection. Once the biopsy was removed, the protocols matched the strict procedures used for the human cardiac surgery RA biopsies, except the courier time was longer.

### 2.2 Porcine study design

All the animal procedures were undertaken at the University of Bristol large animal facilities. All procedures were approved by University of Bristol Research Ethics committee and performed in accordance with the Guide for the Care and Use of Laboratory Animals,^34^ the United Kingdom Animal (Scientific Procedures) Act, 1986, and conform to the guidelines from Directive 2010/63/EU of the European Parliament on the protection of animals used for scientific purposes. Procedural aspects were as previously reported.^35^ Briefly, juvenile, 5–6 months old female Landrace White pigs (weight range 45–75 kg) were subject to general anaesthesia (pre-medication with ketamine, i.m. 15–20 mg/kg, induction with propofol i.v. 16–20 mg/kg and maintained with isoflurane in oxygen with the vaporizer set at 2%).

#### 2.2.1 Atrial appendage study

Animals recruited in the study were undergoing cardiac surgery with CPB procedures (PPL 30/2854) from which RA biopsies were collected following median sternotomy and IV heparin (target INR >400 s) prior to CPB.

#### 2.2.2 Left ventricle study

Animals recruited in the study were from within a control group of pigs undergoing cardiac surgery with CPB procedures (PPL 30/3064 and PPL 7008975) from which LV biopsies from the endocardial surface could be rapidly collected at termination.

All biopsies were at least 1 cm^2^. At the completion of each surgical protocol, and while still under general anaesthesia and full monitoring, animals underwent median sternotomy to expose the heart, the aorta, and both superior and inferior vena cava. Euthanasia involved cardioplegic arrest using cold (0–4˚C) crystalloid solution in the aortic root after aortic clamping and concomitant exsanguination via the inferior vena cava. All porcine biopsies were collected, packed, and promptly couriered to Oxford using the same approach used for human RA and LV biopsies.

### 2.3 Human and porcine sample collection, transfer, and dissection

Once in the laboratory, each human or porcine sample was transferred to a chilled dissecting dish (10°C) containing MOPS buffer, opened and pinned for dissection of IMCAs from within trabeculae (pectinate or papillary muscle) or the subendocardial tissue, respectively, under magnification. For all experiments, IMCAs were cannulated onto glass pipettes (outer diameter up to 100 µm), and tied with 11/0 sutures (Ethicon). The temperature was slowly raised to 36.6 ± 0.3°C, before pressurizing arteries to 80 mmHg using a gravity-fed pressure tower. Each IMCA was longitudinally stretched (final length usually 800–1200 µm), and left to equilibrate for 60 min. After checking for leaks,^36^ only IMCAs with <5% deflation in 1 min, or <5 µL/h were used further. As such, all IMCAs in the study were first assessed for MT, before assessment of endothelial cell (EC) function (vasodilation to bradykinin) and/or SMC constriction (to isotonic KCl). Whilst still pressurized, each artery was then processed for either live/dead stains, EC/SMC Ca^2+^, immunohistochemistry (phalloidin or phenotype), and/or electron microscopy (EM) as shown in *Figure 1*. Within this study, we link the percentage MT generated in each IMCA to specific SMC tests of viability, with the aim of establishing why some arteries are no longer able to develop MT.

### 2.4 SMC Test 1: functional vascular reactivity studies

Before testing function, the hydraulic conductivity (an index of vascular leakiness) of IMCAs was calculated from the volume of fluid leaving the arterial lumen over 1–5 h at 80 mmHg luminal pressure together with the arterial length and inner diameter, as previously.^36^ The first test of SMC contractile function was to establish the extend of MT that developed in response to 80 mmHg luminal pressure, relative to the maximum diameter of arteries. The outcome of this test established how the experiment then progressed, according to the flow chart depicted in *Figure 1*. Only those IMCAs with ≥10% MT were considered to pass this first test, and were used for assessment of endothelium-dependent dilation to bradykinin (0.1 fmol/L to 1 µmol/L). SMC function was further assessed in a sub-group of arteries, which either passed or failed SMC Test 1, as the vasoconstriction to 45 mmol/L isotonic KCl (added to the superfusion). The striking contractile behaviour of h-RA-IMCAs was depicted by the direction of constriction, whether it was radial (circumferential, to reduce diameter), longitudinal (with no clear change in diameter but clear inward, usually twisting, movement) or both together to give a ‘concertina-type’ twisting motion. The longitudinal movement could also be observed as lengthening during dilation, often before the onset of radial dilation. Hence, both the development and reversal of MT were assessed to categorize arteries. Interestingly, the structure of the artery wall could be seen in the transmitted images, and the longitudinal movement was often associated with the presence of abnormal ‘reefs’ of cells within the lumen. Values are the mean ± SEM of *n* patient or pig samples, one artery per sample.

### 2.5 SMC Test 2: 3D structure using confocal fluorescence microscopy

The high incidence of arteries that failed SMC Test 1 suggested the SMCs were either not viable or their arrangement was disrupted. We first tested their arrangement. In SMC Test 2, the orientation of SMCs was established following fixation of cannulated IMCAs at 80 mmHg, using 2% (wt/vol) paraformaldehyde for 10 min at 36.6 ± 0.3°C, then washing with phosphate-buffered saline (PBS). Following fluorescence labelling with phalloidin or antibodies, arteries were imaged while still cannulated and pressurized to 80 mmHg, *z*-stacks obtained using a ×40 objective (1.15 NA, Olympus). Acquired images were colour-coded using Imaris software (version 8.0.2, Bitplane). The unexpected presence of l-SMCs prompted a more detailed investigation aimed at establishing whether their presence helped, hindered, or had no effect on the ability to generate MT as shown in SMC Test 1.

### 2.6 SMC Test 3: 3D structure using EM

SMC Test 2 was taken a step further by performing high resolution imaging to evaluate the cell types present in the arterial wall, and the extent of homo- and heterocellular cell–cell contacts between them. IMCAs processed for EM were fixed at the end of functional assessment for MT and vasomotor responses. Cannulated IMCAs maintained at 80 mmHg and heated to 36.6 ± 0.3°C were fixed, transferred to a vial of buffered fixative, and fixed overnight before processing for transmission electron microscopy (TEM) and serial block face SEM. Data were examined and segmented using Imaris software (version 8.0.2, Bitplane).

### 2.7 SMC Test 4: cell viability studies

Conventional approaches to test the viability of SMCs (and ECs) commenced by assessing their ability to uptake and de-esterify the fluorescent dye calcein AM and by comparing labelling with cell permeant and impermeant nuclear dyes.^37^ Cells were classified as live (calcein in cytoplasm; nuclei stained with Hoechst 33342 but not PI) or dead (nuclei stained with both Hoechst 33342 and PI; no calcein); live cells were expressed as a percentage of all cells in a given image field. Image *z*-stacks were colour-coded using Imaris software (version 8.0.2, Bitplane).

### 2.8 SMC Test 5: intracellular Ca^2+^ studies

The high incidence of arteries that passed SMC Test 4, but not SMC Test 1, required further investigation. After establishing the vaso-reactivity of arteries (SMC Test 1), changes in arterial SMC and/or EC intracellular Ca^2+^ were imaged using the fluorescent Ca^2+^ indicator fluo-8. This relied on viable cells to de-esterify the dye, analogous to SMC Test 4, but also reported whether Ca^2+^ influx and release pathways were operational, and in the case of human arteries, whether contraction occurred. To facilitate dye retention in cells experiments were performed at 31–33°C. Agents were added directly to a static bath, except for KCl, which was rapidly switched to a warmed isotonic KCl solution. Porcine IMCAs retained some MT at 31–33°C, and further contracted to isotonic KCl and caffeine, therefore nifedipine (1 µmol/L) was present to reduce movement, which precluded the use of KCl in these arteries. Responses to both caffeine and bradykinin, to activate Ca^2+^ release via ryanodine receptors (RyRs) and inositol trisphosphate receptors, respectively, were obtained in the same cells, and these agents were used to distinguish longitudinally arranged SMCs (l-SMCs) from ECs, which was further confirmed by the Ca^2+^ influx in response to depolarization with isotonic KCl. Values are summarized as the mean ± SEM, with *n* representing the number of arteries studied.

### 2.9 SMC Test 6: contractile phenotype studies

Neither patient history nor the first five SMC Tests clearly identified why nearly 50% of arteries did not develop ≥10% MT. Some arteries that appeared viable did not contract, even if SMC Ca^2+^ signalling was evident. Therefore, experiments were performed to assess the phenotype of SMCs in IMCAs within and across species. Importantly, the contractile and synthetic marker antibody concentrations, online confocal imaging, and offline image processing were kept as constant as possible. The image acquisition settings were consistent within the 3D *z*-stack, allowing intra-artery comparison of radial SMC (r-SMC) and l-SMC expression of SM-MHC or α-SMA, alongside caldesmon or vimentin. These settings were then matched as closely as possible for all arteries studied. Arteries were imaged such that the *x*–*y* axis ran along the length of the artery, which allowed multiple r-SMCs to be visualized in a given field (up to ∼30 r-SMCs). Multi-channel image *z*-stacks (contractile and synthetic markers, plus nuclei and EC label) were analysed offline using Imaris software (version 8.0.2, Bitplane). This allowed counts of individual r-SMCs with greater relative intensity for synthetic vs. contractile markers within their cytoplasm as a percentage of all r-SMCs visible in the field, providing values for percentage synthetic r-SMCs. This approach was considered the most informative and accurate index of relative marker expression within and between arteries. Separately, to calculate the percentage of cells expressing caldesmon in r-SMC to l-SMC, the average fluorescence intensity of lines drawn across either r-SMCs or l-SMCs were used, in most cases from a plane where both r-SMCs and l-SMCs were clearly visible and separated. The same lines could be used for both contractile and synthetic markers within a given image plane. This enabled both a direct comparison of the same protein between r-SMCs and l-SMCs, and an indirect comparison between contractile and synthetic proteins.

### 2.10 Materials

NaCl, KCl, and D-glucose were purchased from Fisher Scientific (Loughborough, UK); MOPS, EDTA, CaCl_2_ 2H_2_O, pyruvate, PBS sachets, Tween20, and bovine serum albumin were purchased from Sigma (Poole, UK); MgSO_4_ 7H_2_O, NaOH, and NaH_2_PO_4_ H_2_O were purchased from VWR (Leicestershire, UK); and bradykinin was purchased from Tocris (Bristol, UK). The cellular dyes Hoechst 33342 (H3570), PI (P1304MP), DAPI (D3571), and AF-633 (A30634) were purchased from ThermoFisher Scientific (Paisley, UK), phalloidin-TRITC from Sigma-Aldrich (Dorset, UK), and fluo-8 AM from AAT Bioquest (Stratech, Newmarket, UK). Paraformaldehyde was purchased from Electron Microscope Sciences (Hatfield, PA, USA).

### 2.11 Statistics

Statistical analysis was performed using GraphPad Prism software (version 8, GraphPad Software, La Jolla, USA), where *P* < 0.05 was considered significant. All specimens collected were analysed and no experimental data were excluded from the study. Formal statistical comparisons on our unpaired data first tested for Gaussian distributions (D’Agostino and Pearson omnibus normality test), which confirmed that for all comparisons non-parametric tests should be used with no assumption of equal standard deviation. Subsequently, two groups were compared with two-tailed Mann–Whitney tests, and groups of three or more with Kruskal–Wallis tests and Dunn’s post-test. For this analysis values are the mean ± SEM. To correlate MT results with synthetic markers in r-SMCs or with other variables, univariable and multivariable linear regression models were run using R (version 3.6.0, https://www.R-project.org/) and jtools (version 2.0.1, https://cran.r-project.org/package=jtools), and as indicated, adjusted for any known risk factors (Model A: sex, type of valve disease, hypertension, hypercholesterolaemia, and smoking history) and medications (Model B: statins, diuretics, Ca^2+^ channel blockers, beta-blockers, aspirin, anticoagulants, and angiotensin-converting-enzyme-inhibitors). Multiple regression linear models were obtained after a forward/backward stepwise selection process with AIC as the selection criterion for the final model. Analysis for a global validation of the linear model assumptions as well as separate evaluations of skewness, kurtosis, link function, and heteroscedasticity were tested using R (package, lmSupport; function, modelAssumptions) and all were found to be acceptable. Original data points are presented as scatter plots where possible, and these data were tested for Gaussian distributions (D’Agostino and Pearson omnibus normality test or Shapiro Wilk test). For ease of visualization, the mean ± SEM are shown on the scatter plots. The inclusion of all arteries, including those that did not develop MT, meant the 88 MT values for h-RA-IMCA data were not normally distributed, so we report the median with lower 95% and upper 95% confidence intervals in the figure legends using the format [median, LCI, UCI]. Other data were normally distributed and, where reported, the median was shown as [median].

## 3. Results

This study reports the outcomes of assessing pressure-induced MT for each IMCA studied from every biopsy transported to the laboratory, without exclusion. We commenced our succession of protocols using surgically provided biopsies (h-RA-IMCAs) (*Figure 1A–C*) and later extended these to include (i) RA and LV IMCAs from organ donor procedures (hOD-RA-IMCAs and hOD-LV-IMCAS); and (ii) porcine IMCAs (p-RA-IMCAs and p-LV-IMCAs).

### 3.1 MT of h-RA-IMCAs

**Figure 2:**
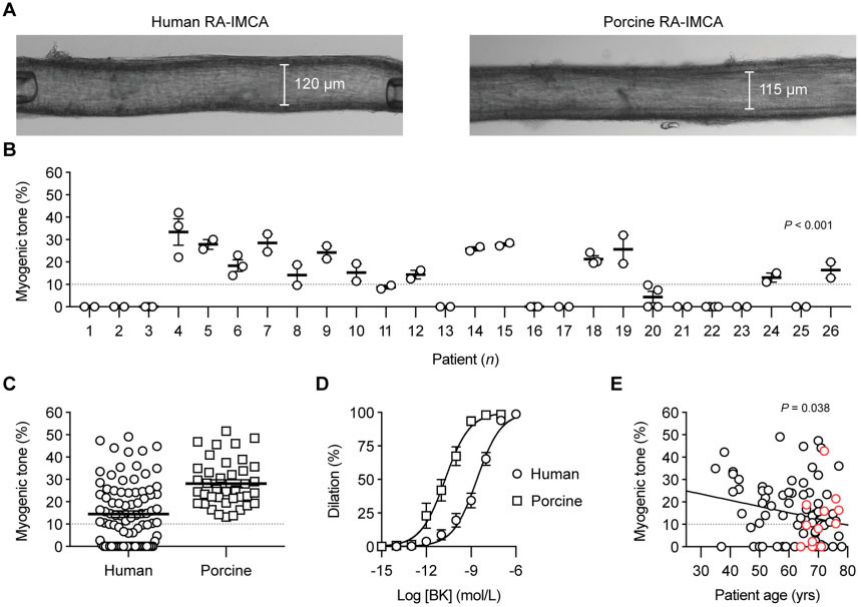
Vaso-reactivity of human and porcine RA-IMCAs. (*A*) Micrograph of an isolated, cannulated and pressurized h-RA-IMCA and p-RA-IMCA with MT. (*B*) The MT developed in h-RA-IMCAs within each patient biopsy was consistent, but varied between biopsies (*n* = 26; non-parametric Kruskal–Willis test with Dunn’s post-test). If an intra-biopsy mean MT was ≥10% it was different to the mean for Patient 1. (*C*) Summary of percentage MT in h-RA-IMCAs [(12.5%,8.6,16.3), *n* = 88] and p-RA-IMCAs [(25.3%), *n* = 39]. (*D*) Concentration-dependent vasodilation to bradykinin (BK), assessed from developed MT, in h-RA-IMCAs (*n* = 33) and p-RA-IMCAs (*n* = 19). (*E*) Relationship between patient age and MT (*n* = 88, red circles indicate 12 patients on calcium channel blockers). One-way ANOVA in (*B*), and linear regression in (*E*). Further details regarding patient demographics and medications available in *Table 1* and Supplementary material online, *Figure S1A–D*.

Following our assessment of MT using confocal microscopy (SMC Test 1) (*Figure 2A*), all the subsequent experimental protocols were successfully performed using the cannulated arteries. On 26 occasions, multiple h-RA-IMCAs were dissected from the same biopsy/patient, allowing an intra-sample comparison of MT. We found that measurements were consistent across multiple arteries from the same biopsy, thus suggesting that each artery faithfully representing the condition of the patient sample (*Figure 2B*). The average pressure-induced MT in h-RA-IMCAs was 14.5 ± 1.5% (*n* = 88, from passive diameter, 135 ± 4 µm) (*Figure 2C*) . About 56% of h-RA-IMCAs developed ≥10% tone (24.3 ± 1.5% tone, *n* = 49) and, from this vaso-reactive group, EC-dependent dilation to bradykinin was established (pEC_50_ = 8.6 ± 0.1, *n* = 33) (*Figure 2D*). A univariable analysis showed a significant, but weak, correlation of poor MT with ageing (*P* = 0.038) (*Figure 2E*); however univariable and multivariable models adjusted for other risk factors and medications showed no clear correlation of disease circumstance with poor MT (Supplementary material online, *Figure S1* and *Table S1*).

### 3.2 Tissue architecture and 3D cell alignment in h-RA-IMCAs

**Figure 3:**
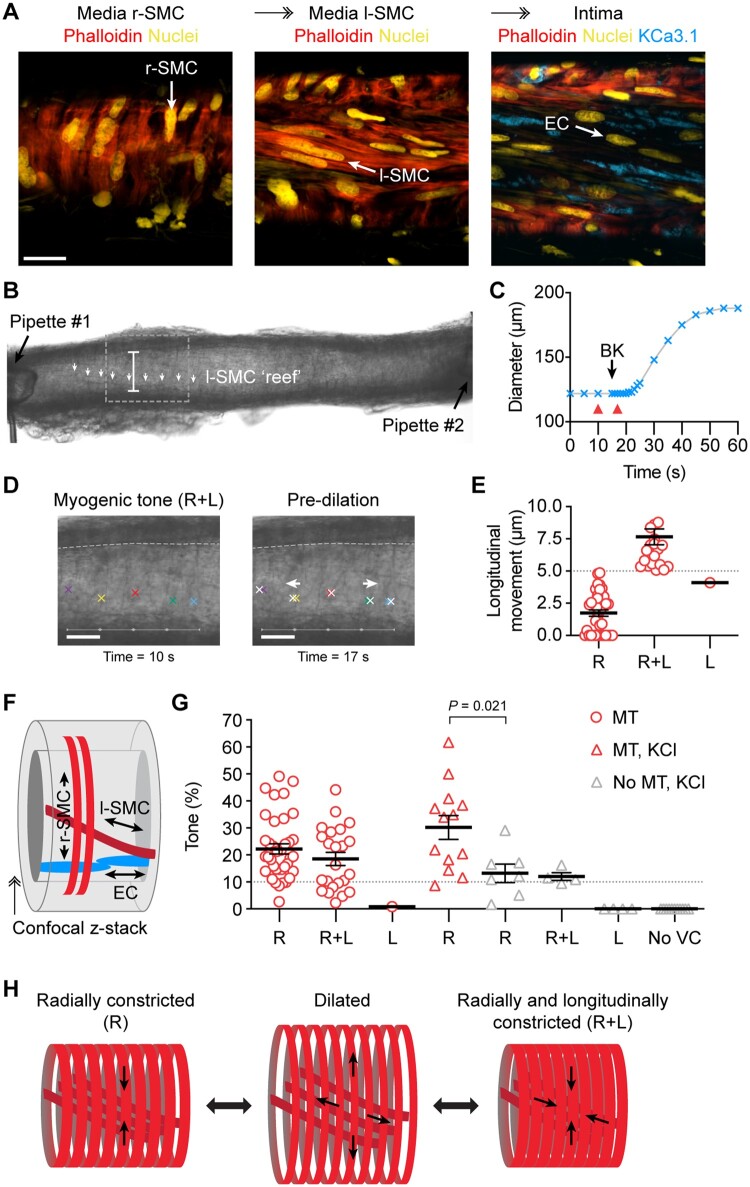
Longitudinally aligned medial SMCs (l-SMCs) can cause longitudinal movement in h-RA-IMCAs. (*A*) Confocal micrographs from a cannulated and pressurized h-RA-IMCA. The confocal z-stack reveals circumferentially arranged r-SMCs and l-SMCs stained with phalloidin, and further towards the lumen in the same artery, K^+^-channel (K_Ca_3.1) label of ECs; bar =20 µm. (*B*) Micrograph of an isolated, cannulated and pressurized h-RA-IMCA with MT, with cannulating pipettes visible. Careful inspection of the wall reveals a diagonally orientated sheet or ‘reef’ of l-SMCs (white arrows); bar =122 µm. (*C, D*) The artery in (*B*) was treated with 1 µmol/L BK to generate a time-course of dilation (*C*). (*D*) Frame-by-frame analysis of the motion revealed longitudinal (L) movement of the wall before the onset of radial movement; red arrowheads in (C*)* refer to images in (*D*), white crosses refer to movement; bars =50 µm. After a few seconds, the wall began to dilate radially with an ultimate diagonal ‘concertina-type’ twisting effect. Based on this motion, the MT in this artery was classified as ‘R+L’ constriction. (*E*) The magnitude of longitudinal motion was assessed for every artery (*n* = 88). Only arteries where clear longitudinal movement of ≥5 µm were considered R+L. (*F*) Schematic depiction of the cellular arrangement. (*G*) Most h-RA-IMCAs developed MT in a radial manner (R, *n* = 38), including some with additional longitudinal movement (R+L, *n* = 23), and one artery with clear longitudinal movement (L) but no change in diameter. The vasoconstrictor tone to isotonic 45 mmol/L KCl (KCl) was assessed in a subset of arteries with MT (red triangles, *n* = 13) and without MT (grey triangles, *n* = 27). Non-parametric unpaired *t*-test, Mann–Whitney post-test. (*H*) Schematic depicting the dilated state and the two most commonly observed modes of SMC contraction, either R or R+L. ↠, Confocal *z*-stack through the wall of the IMCA.

Ultrastructural evaluation of h-RA-IMCA SMC density and orientation (SMC Test 2) (*Figure 3A*) showed the typical circumferentially orientated SMCs responsible for radial contraction (r-SMCs). Shifting the focal plane towards the endothelium revealed an additional 3D layer of longitudinal/spiral SMCs bridging r-SMCs and ECs, which we name ‘longitudinal-SMCs’ (l-SMCs) (*Figure 3A*), before reaching the ECs. l-SMCs were visible in transmitted light (*Figure 3B*), and slight movement of the wall in the longitudinal axis (5–10 µm) could be observed and measured within the confines of fixed position pipettes at each end of arteries (*Figure 3B–E*). This inner layer of l-SMCs (*Figure 3F*) was therefore contractile in some arteries. Evaluation of arterial motion during measurements of MT or vasoconstrictor responses to isotonic KCl revealed that 51.1% of h-RA-IMCAs visibly contracted solely along the circumferential/radial axis; 30.7% additionally contracted along a longitudinal/twisting axis; 5.7% only moved in a longitudinal/twisting axis; and 12.5% were completely vaso-inactive (*Figure 3G*). Overall, the r-SMC and l-SMC layers appeared linked together into a ‘concertina-type’ 3D tissue architecture within the h-RA-IMCA (*Figure 3H*).

### 3.3 Spatial 3D resolution of ECs, l-SMCs, and extracellular matrix

**Figure 4:**
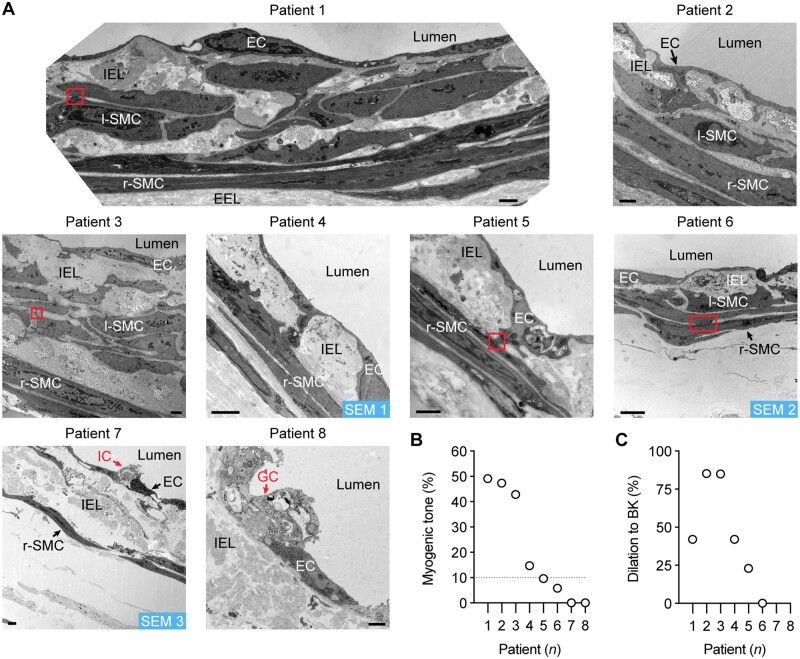
h-RA-IMCA MT and EC-dependent vasodilation linked to high resolution structure. (*A*) TEM from cannulated and pressurized h-RA-IMCAs. Images show multiple sites of contact between ECs and SMCs (r-SMC and l-SMC). Severely damaged ECs (ghost cells) remained in close contact with adjacent ECs. Inflammatory cell; bar =1 µm. l-SMCs, when present, are always found between the r-SMC and EC layers. SEM 1–3, indicates h-RA-IMCAs processed for SBF-SEM (Supplementary material online, *Figure S3A*). The level of MT (*B*) and vasodilation to 10 nM BK (*C*) in each h-RA-IMCA subsequently processed for EM (Patients 1–8) reveals arterial ultrastructure is markedly altered in arteries without MT, with extensive thickening of the elastin layer separating the ECs and SMCs (IEL).

A longitudinal internal elastic lamina (IEL) was observed abluminal to the endothelial monolayer in p-RA-IMCAs (Supplementary material online, *Movie S1*). In h-RA-IMCAs, a high density of elastin could also be seen between and beyond SMCs (*Figure 4A* and Supplementary material online, *Figure S2*). Nevertheless, the ability of arteries to distend to luminal pressure did not predict whether MT could develop (Supplementary material online, *Figure S2D*). TEM revealed h-RA-IMCAs that developed MT had uniformly arranged r-SMCs, whereas the spatial arrangement of r-SMCs in those that did not develop tone was more intermittent. l-SMCs were identified, making multiple cell–cell contacts with r-SMCs and/or ECs (SMC Test 3) (*Figure 4A*) (Patients 1–3 and 6). In the same h-RA-IMCAs, the extent of MT was generally associated with EC function (*Figures 4B and C*). To extend our observations, the 3D spatial structure and cell–cell contacts in 3 of the 8 h-RA-IMCAs were revealed using serial block face-scanning electron microscopy (SBF-SEM) (Supplementary material online, *Figure S3A* and *Movies S2–S4*). Combining all imaging approaches, we found that l-SMCs were always evident as small groups or ‘reefs’ and were present in 66% of h-RA-IMCAs (*n* = 32). Of the 32 arteries within which the arrangement of SMCs was imaged, there was no clear link between the presence of l-SMCs and the ability to develop MT. The developed MT was similar in RA-IMCAs with (11.3 ± 3.8% tone, *n* = 21) or without l-SMCs (8.9 ± 2.8% tone, *n* = 11).

### 3.4 Cell viability and Ca2^±^ signalling in h-RA-IMCAs

Measurements of cell viability in h-RA-IMCAs indicated that there was no correlation (*P* = 0.232) between the extent of MT and percentage of viable r-SMCs (SMC Test 4, Supplementary material online, *FigureS4A–C*). Even in IMCAs with no MT, 70 ± 6% (*n* = 7) of cells were viable. ECs were also predominantly viable (83 ± 4%, *n* = 12) (Supplementary material online, *FigureS4A andB*), and able to drive EC-dependent dilation (Supplementary material online, *Figure S4D*). Ca^2+^ indicator dyes further defined viable cells and each cell type (SMC Test 5, Supplementary material online, *Figure S4E*). Analysis of Ca^2+^ indicator fluorescence responses over time showed h-RA-IMCAs with poor MT (5.6 ± 2.4%, *n* = 12, including 7 with no MT) increased intracellular Ca^2+^ to isotonic KCl and caffeine, and ECs responded to bradykinin (Supplementary material online, *Figure S4F*). This demonstrates that viable SMCs with functional voltage-dependent Ca^2+^ channels and RyRs do not necessarily develop MT. Finally, to test water barrier function, hydraulic conductivity measurements indicated no difference between h-RA-IMCAs with MT (44 ± 11 ×10^−7^cm/s/cmH_2_O, 29 ± 5% tone, *n* = 5) and those with <10% MT (43 ± 7 ×10^−7^cm/s/cmH_2_O, 3 ± 1%, *n* = 8).

### 3.5 Lack of MT correlates with synthetic phenotype in h-RA-IMCA r-SMCs

**Figure 5:**
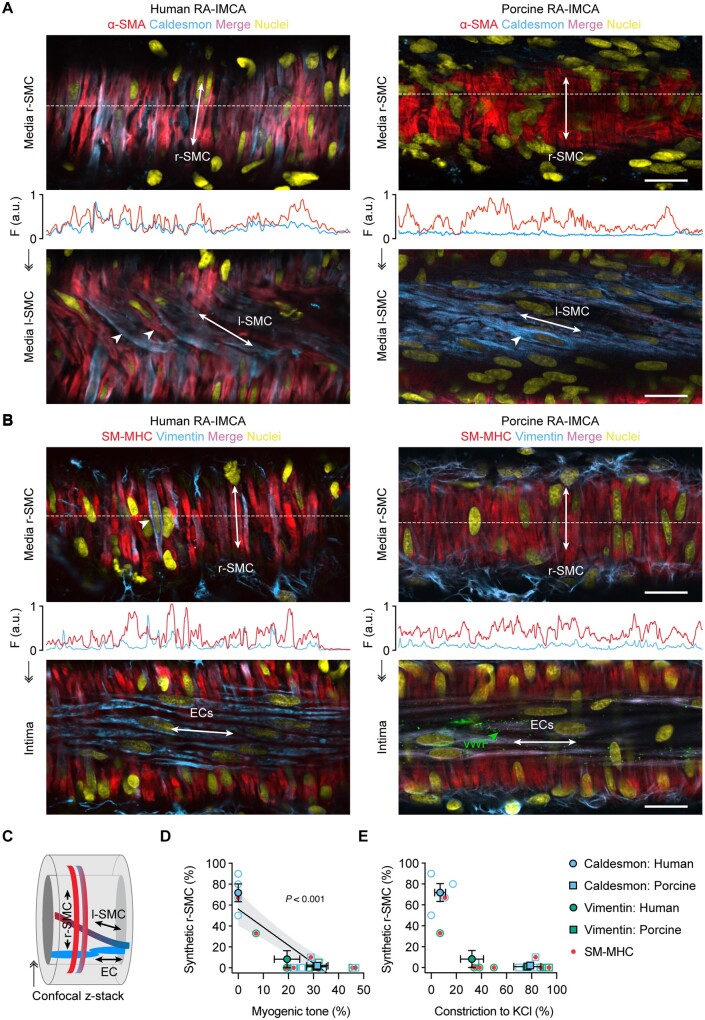
The contractile phenotype of r-SMC in h-RA-IMCAs and p-RA-IMCAs correlates with vasomotor function. (*A* and *B*) Confocal micrographs from cannulated and pressurized IMCAs. Each artery was imaged at two focal planes: towards the outer wall to image r-SMCs, and towards the lumen to image l-SMCs or ECs [indicated in (*C*)]. (*A*) r-SMC and l-SMCs labelled for α-SMA and caldesmon (see Supplementary material online, Movies S6 and S7). Note the heterogeneity of α-SMA labelling in h-RA-IMCA l-SMC (arrowheads). (*B*) Cell labelling for SM-MHC and vimentin. Vimentin was sparse in r-SMC, while homogeneous in both ECs (also indicated by von Willebrand Factor, vWF) and perivascular cells (fibroblasts). (*A* and *B*) The labelling profile for each protein is indicated by the fluorescence intensity (F, arbitrary units, a.u.) of a line drawn through the image at the point indicated by the white dashed lines. Bar =20 µm. Representative of three human and three porcine arteries. (*D* and *E*) Percentage of r-SMC with relatively stronger expression of synthetic markers (caldesmon or vimentin) than contractile markers (SM-MHC or α-SMA) within the cytoplasm of the cell plotted against the percentage of developed MT generated in each artery (*D*); and in the same arteries, the percentage vasoconstriction to 45 mmol/L isotonic KCl (*E*). Open symbols, individual data points; closed symbols, mean data; red dots, SM-MHC antibody. Linear regression through individual data points, with 95% confidence interval, in (*D*). ↠, Confocal *z*-stack through the wall of an IMCA.

The disconnect between contractile dysfunction in SMC Test 1 and viability (SMC Tests 2–5) required another explanation for the lack of MT in almost 50% of h-RA-IMCAs. The evidence pointed downstream of Ca^2+^ handling, so SMC phenotype was interrogated (SMC Test 6). Immunolabel for the contractile proteins SM-MHC and α-SMA was compared to the synthetic phenotypic markers caldesmon (l-caldesmon isoform)^38^ and vimentin. A gross heterogeneity in expression of SM-MHC or α-SMA relative to caldesmon was observed in both r-SMCs and l-SMCs, with individual SMCs often differing from neighbour SMCs (*Figure 5A* and Supplementary material online, *MovieS6*). A similar profile was observed with SM-MHC or α-SMA relative to vimentin, although vimentin also labelled ECs and fibroblasts (*Figure 5B*). This cell-specific immunolabelling also confirmed the presence of l-SMCs between the ECs and r-SMCs (*Figure 5A and C*). Poor MT was associated with caldesmon or vimentin expression in the r-SMC cells (*Figures 5D and E*) in this 59–70 years old group of patients (*n* = 8, *P* = 0.003).

### 3.6 MT, tissue architecture, cellular phenotyping, and function of hOD-LV-IMCAs

**Figure 6:**
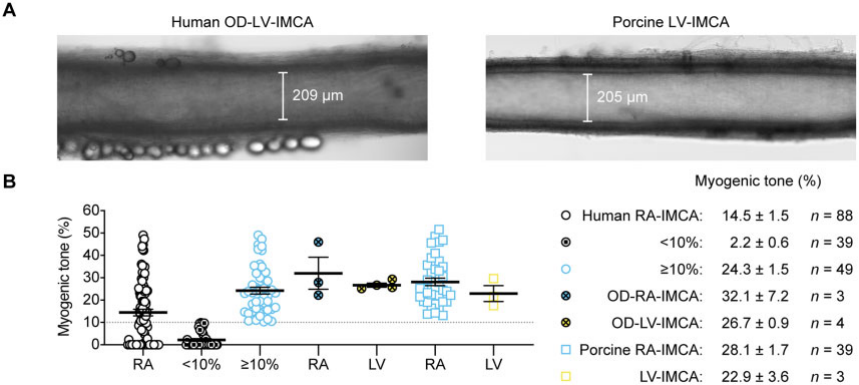
Summary of MT development in *ex vivo* human and porcine IMCAs isolated from RA appendages and the endocardial surface of left ventricles. (*A*) Micrograph of an isolated, cannulated and pressurized human organ donor (OD)-LV-IMCA and porcine LV-IMCA with MT. (*B*) When data from the 88 human RA-IMCAs were divided into two groups such that those with <10% MT were shown separately [(0.0%,0.0,0.9), *n* = 39], the human RA-IMCA group with ≥10% MT [(23.2%), *n* = 49] appeared remarkably similar to the organ donor [(27.9%), *n* = 3, (26.2%), *n* = 4] and porcine [(25.3%), *n* = 39, (21.6%), *n* = 3] RA- and LV-IMCA groups, respectively. The human RA-IMCA ≥10% group represents human RA-IMCAs generally used by researchers for vaso-reactivity studies, whereas the human RA-IMCA <10% group represent RA-IMCAs with microvascular contractile dysfunction. The mean±SEM values are provided for each group. Note that, all arteries were studied *ex vivo* under the same conditions by the same research team. Data from *Figure 2C* are included, for comparison.

**Figure 7:**
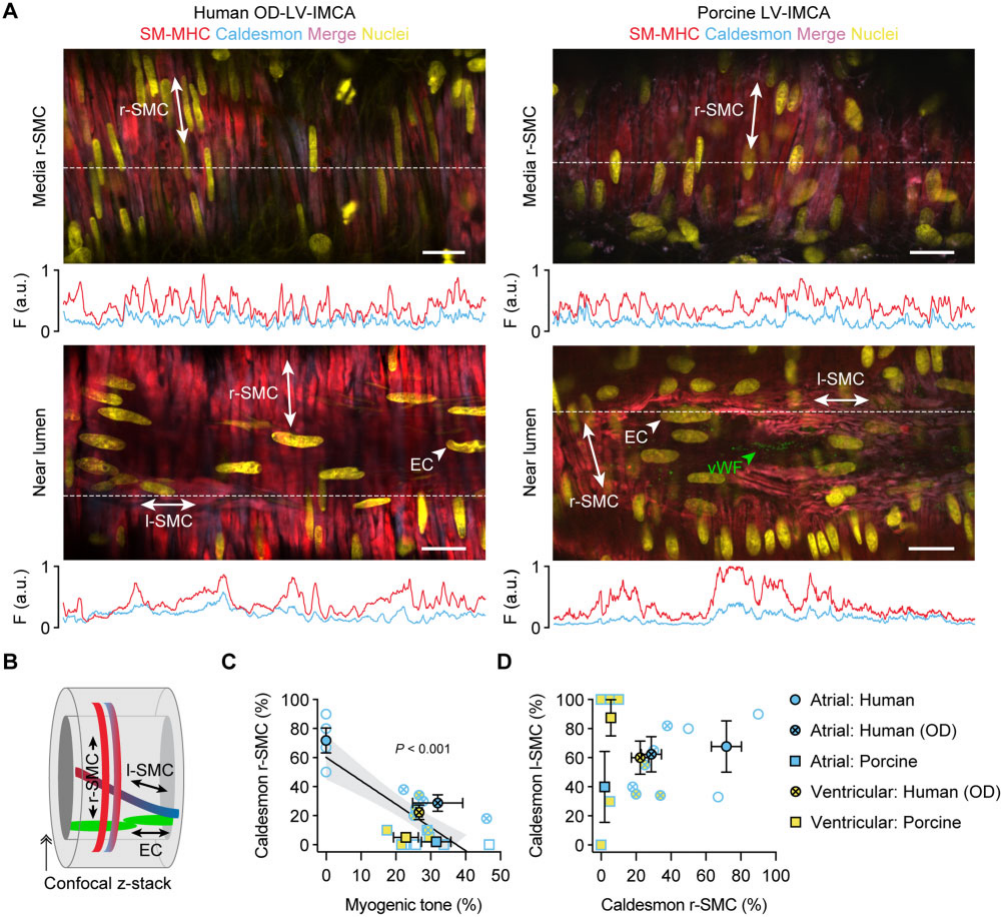
The contractile phenotype of r-SMC in both human and porcine IMCAs correlates with vasomotor function. (*A*) The LV-IMCAs shown in *Figure 6A* were labelled for SM-MHC and caldesmon and r-SMCs, l-SMCs and ECs imaged (schematic in *B*). The position of ECs was confirmed using label for vWF. The labelling profiles for each protein are indicated by the fluorescence intensity (F, arbitrary units, a.u.) of a line drawn through the image at the point indicated by the white dashed lines. Representative of 4 human OD and 11 porcine LV-IMCAs. (*C*) Percentage of r-SMC with relatively stronger expression of caldesmon than SM-MHC within the cytoplasm of the cell plotted against the percentage developed MT in each artery. All data using this combination of markers is shown from both atrial and ventricular biopsies from both species and heart chambers, for comparison. In general, the human OD-LV-IMCAs had more heterogeneous labelling of contractile markers than the pig LV-IMCAs despite similar levels of developed MT (see *Figure 6*). Of 4 human OD-LV-IMCAs, 3 had visible l-SMCs (75%), and of 11 porcine LV-IMCAs, 8 had visible l-SMCs (73%). (*D*) Percentage of cells with clear caldesmon expression in l-SMC plotted against percentage of cells with expression in r-SMCs (*n* = 3 human, and *n* = 8 porcine LV-IMCAs). Open symbols, individual data points; closed symbols, mean data. Linear regression through individual data points, with 95% confidence interval, in (*C*). ↠, Confocal *z*-stack through the wall of an IMCA.

To ascertain the relevance of our findings using h-RA-IMCAs to h-LV-IMCAs, and given it was unethical and hence not possible to obtain large ventricular biopsies from cardiac surgery procedures in Bristol, we sourced a small number of human LV (and RA) biopsies (∼1 cm^2^) from participants subjected to organ donor (hOD) procedures in Newcastle. Using the same methodological approach, we were able to obtain a small number of human atrial IMCAs (hOD-RA-IMCAs) and ventricular IMCAs (hOD-LV-IMCAs). The aim here was to compare SMC Test 1 to SMC Test 6 to check the validity of our finding that MT related to SMC phenotype, and could be translated across heart chambers. hOD-IMCAs were successfully cannulated and each developed good MT (≥10%) in response to 80 mmHg luminal pressure (*Figures 6A and B*). When comparing h-RA-IMCAs exhibiting ≥10% MT in *Figure 2* to hOD-RA-IMCAs and then to hOD-LV-IMCAs, the level of MT development was remarkably similar (*Figure 6B*). Human OD-LV-IMCAs had high levels of SM-MHC in r-SMCs (*Figure 7A*, orientation *Figure 7B*), with similar profiles of expression between heart chambers (*Figure 7C*). We were also able to confirm that l-SMCs are present in both heart chambers, regardless of human tissue source (*Figure 7A and D*).

### 3.7 Porcine IMCAs as a relevant preclinical model

SMC Tests were also performed using porcine RA-IMCAs. p-RA-IMCAs developed ≥10% MT (from passive diameter 202 ± 12 µm, *n* = 39), with an average of 28.2 ± 1.7% MT (SMC Test 1) (*Figure 2C*). EC-dependent dilation to bradykinin was ∼100× more sensitive than in h-RA-IMCAs (pEC_50_ = 10.7 ± 0.1, *n* = 19) (*Figure 2D*). Combined data from confocal fluorescence microscopy images showed that 82% of p-RA-IMCAs (*n* = 22) featured l-SMCs (one example in *Figure 5A*), which were confined to sub-sections of the IMCAs (Supplementary material online, *Figure S3B*). Despite this high incidence of l-SMCs, 95% of p-RA-IMCAs (*n* = 39) featured radial contraction, with no detectible longitudinal/twisting motion. The porcine arterial SMCs and ECs showed changes in cytosolic Ca^2+^ similar to that observed in h-RA-IMCAs (SMC Test 4, Supplementary material online, *Figure S4F*). The hydraulic conductivity of p-RA-IMCAs was 13.9 ± 3.8 ×10^−7^cm/s/cmH_2_O (*n* = 4).

The co-expression of SM-MHC or α-SMA and caldesmon in p-RA-IMCAs differed between r-SMCs and l-SMCs. r-SMCs from p-RA-IMCAs uniformly expressed SM-MHC and α-SMA but almost no caldesmon; and while l-SMCs also expressed SM-MHC and α-SMA, caldesmon was clearly evident (SMC Test 6) (*Figure 5A* and Supplementary material online, *Movie S7*). A similar profile to caldesmon was observed with vimentin in r-SMCs (*Figure 5B*); the major difference being that vimentin was not observed in l-SMCs, despite clear labelling with SM-MHC or α-SMA and positive detection of vimentin in ECs and fibroblasts (*n* = 3). In p-RA-IMCAs, robust MT and constriction to isotonic KCl were associated with almost no expression of caldesmon or vimentin in r-SMCs (*Figure 5D and E*). Univariable analysis of the combined human and porcine RA-IMCA data showed a significant correlation of synthetic markers in r-SMCs with poor MT (*P* < 0.001) (*Figure 5D*).

Finally, when these phenotypic data were plotted against developed MT in both species, cardiac chambers and sources of human tissue, the relative expression of caldesmon and SM-MHC in r-SMCs strongly correlated with poor MT (*P* < 0.001) (SMC Test 6) (*Figure 7*).

## 4. Discussion

The key finding of this study is the demonstration that poor MT observed in h-IMCAs isolated from non-ischaemic human myocardium is associated with a synthetic phenotype in still viable r-SMCs. As such these usually discarded arteries form a novel model for studying coronary microvascular contractile dysfunction. In addition, by using organ donor tissue, we validate the findings obtained from h-RA-IMCAs and extend to IMCAs from the human left ventricle. The trends in data were supported by atrial and ventricular arteries studied from pigs, in doing so providing a clinically relevant large animal model to study microvascular function. Overall, the comparisons of RA- vs. LV-IMCAs in humans and in pigs showed equivalent links between MT and the synthetic phenotype of SMCs, 3D tissue architecture, and cell viability.

RA-IMCAs provide the primary source of human *ex vivo* live-cell coronary microvascular studies. To date no studies have established why MT does not develop in a subset of IMCAs, these unresponsive arteries are usually discarded or not mentioned.^23–25^ The precious nature of these biopsies means in some cases vasoconstrictor agonists is employed to augment weak MT, but arterial viability is not systematically reported.^28^ These approaches are justified when the focus of the study is based on vaso-reactivity. However, in this study, we intentionally used all IMCAs to identify determinants of poor MT, highlighting the potential relevance of this subset to coronary microvascular dysfunction.

The time delay from tissue harvesting to *ex vivo* testing was minimized for all human and porcine biopsies (h-RA-IMCAs < p-IMCAs ≪ hOD-IMCAs). We are confident that the observed variations in h-RA-IMCA MT, phenotype, function, and 3D architecture are unlikely to arise *ex vivo*. The lack of MT in 44% of h-RA-IMCAs/patients was not due to cell death or poor viability when assessed by live-dead staining or by the ability to raise cytosolic [Ca^2+^] in response to vasoconstrictors. All IMCAs derived from healthy human (including organ donors) or pig developed an average of ∼25% MT following equivalent methodological approaches and timing.

The key finding of this study was the association between the lack of MT in IMCAs and the presence of SMCs with a synthetic phenotype. Defining the best markers to discriminate between synthetic and contractile SMCs is still ongoing in the literature.^1,^^2,^^39–42^ This study shows the ratio of caldesmon to SM-MHC is a combination that fits well with the accepted categorization;^2^ especially since vimentin also labels ECs and fibroblasts, while α-SMA also labels immature or de-differentiated SMCs. It was not necessary to use a selective l-caldesmon antibody; the relative expression of both caldesmon isoforms, when combined with SM-MHC label, very clearly corresponds with SMC contractile function and is therefore highly useful in itself to discriminate between SMC phenotypes. Future studies could use single-cell nuclear-SEQ or spatial transcriptomics to establish whether other markers^2,^^43,^^44^ could also serve as discriminators between synthetic and contractile SMCs within IMCAs subjected to a matched functional assessment of MT development. Our finding that the developed MT in an IMCA from a given biopsy closely represents other IMCAs could aid this difficult question. Ultimately the precise molecular switches to a synthetic phenotype should be identified,^45^ alongside treatments aimed at preserving key extracellular proteins and peptides.^2^

Human RA-IMCAs had dense layers of elastin and collagen between cells, more so than in porcine RA-IMCAs, in keeping with similar findings by others in human and porcine parietal pericardial micro-arteries.^46,^^47^ While we showed no clear relationship between percentage MT and the ability for passive distension, a sub-group of arteries were less able to passively increase diameter to raised luminal pressure. We did not assess the thickening of the extracellular matrix in these arteries. Therefore, we propose that in future experiments, routine measurements of passive distention should be performed, and arteries that are ‘inflexible’ are considered a separate group, not suitable for functional analysis. We also propose that when feasible, fluorescence-based live-cell imaging of the thickening of the extracellular matrix should also be performed,^46,^^47^ and matched to contractile function. Higher resolution imaging using EM has been performed previously in cardiac IMCAs.^48–50^ A distinct advantage of our approach was we fixed the h-RA-IMCAs at physiological luminal pressure following MT measurement. This not only allowed us to image detailed 2D and 3D structure to MT, but also provided novel insights into the arrangement and shape of SMCs as radial or longitudinal along the artery axis. The l-SMCs in the sub-endothelial layer of IMCAs is consistent with reports in large epicardial coronary arteries.^51,^^52^ We found that the l-SMCs intertwine and integrate with the r-SMCs, raising the question as to their source and whether there are parallels with pathological processes observed in epicardial large coronary arteries^53–56^ or vein grafts,^57,^^58^ where recruited SMCs migrate to form an intimal layer.

Comparative evaluations confirmed that atrial and ventricular IMCAs from healthy humans and pigs are equivalent in terms of MT, cell viability and function, phenotypic profile of SMCs, and IMCA 3D architecture. We also noted that if the cut-off for poor MT was set at <10% the average MT in h-RA-IMCAs was similar to hOD-RA-IMCAs and hOD-LV-IMCAs, indicating a valuable index for future studies.

### 4.1 Study limitations

A major limitation of this field is not being able to establish the myogenic reactivity of LV-IMCAs in patients with heart disease either *in vivo* or *ex vivo* and, specifically relevant to this study, those with heart valve disease. This cohort of patients underwent voluntary, recovery surgery, and sufficiently large LV biopsies were not ethically possible to harvest. While it is possible to demonstrate that coronary flow reserve is compromised in patients with heart valve disease,^59^ this cannot be precisely linked to the myogenic reactivity of LV-IMCAs either *in vivo* or *ex vivo*. Therefore, we have made every attempt to address this, albeit indirectly, by demonstrating (i) the RA-IMCAs from organ donors have similar MT development and phenotypic profiles to the subset of RA-IMCAs from valve surgery patients that developed ≥10% MT; (ii) in the same organ donor patients, the myogenic reactivity of the LV-IMCAs was the same in their RA-IMCAs; and (iii) the MT in porcine IMCAs was the same as organ donor IMCAs. Therefore, we can only speculate that the subset of heart valve disease patients with <10% MT in their RA-IMCAs had similarly affected ventricular microvascular arteries. This is clearly an area that warrants much future investigation, the first step being to match measurements of coronary flow reserve *in vivo* to RA-IMCA myogenic reactivity *ex vivo* in an intra-patient context. The ability to routinely obtain RA-IMCAs for *ex vivo* study could then provide a clinically useful surrogate biomarker for ventricular microvascular dysfunction in these patients.

## 5. Conclusions

In conclusion, our data suggest that >40% of h-IMCAs from patients undergoing surgery for valve disease, but without severe coronary disease or pulmonary hypertension, are affected by poor MT and that this finding is strongly associated with a synthetic phenotype of viable r-SMCs. These findings may offer an insight on coronary microvascular dysfunction that may predate the development of clinically indicated heart disease, such as heart failure and warrant further investigations.

## Supplementary material

Supplementary material is available at *Cardiovascular Research* online.

## Authors’ contributions

K.A.D. conceived and designed the experiments, collected and analysed data, prepared the figures, and wrote the manuscript. L.B., X.Y., C.P., T.Z.B., and C.P.S. collected and analysed data. T.S., E.J., and A.P. developed protocols and collected data. N.S. helped with experimental design and interpretation. V.D.B. provided statistical support. R.A. and M.T. provided human and porcine specimens, and contributed to manuscript preparation. All authors proof-read the manuscript.

**Conflict of interest:** none declared.

### Funding

This work was supported by the British Heart Foundation (BHF) and Medical Research Council (MRC) grants to R.A. [grant numbers BHF: PG/18/49/33833, IG/14/2/30991, PG/16/104/32652, and MRC MR/L012723/1], and by the Bristol NIHR Biomedical Research Centre. Organ donor heart collection was funded by the NIHR Cambridge/Newcastle Blood and Transplant Research Unit (BTRU). In addition, this work was supported by British Heart Foundation grants to K.A.D. [grant numbers FS/08/033/25111, FS/13/16/30199, IG/13/5/30431, and PG/18/11/33552], and by the Oxford BHF Centre of Research Excellence [grant number RE/13/1/30181].

## Data availability

The data underlying this article are either incorporated into the article and its Supplementary material online or will be shared on reasonable request to the corresponding author.

## Supplementary Material

cvab218_Supplementary_Data

## Notes

Translational perspective Vasoconstriction of micro-arteries to luminal pressure is a fundamental control on blood flow within the coronary microcirculation, but MT cannot be independently measured *in vivo*. Patients with poor MT would not only have dysregulated microvascular blood flow, but the superimposed problem of limited scope for vasodilation, and hence poor coronary flow reserve. By moving to *ex vivo* studies, our finding that >40% of heart valve disease patients have arteries with poor MT identifies an *ex vivo* model to study human microvascular contractile dysfunction and will stimulate research to assess associations with other biomarkers and their impact on long-term patient outcome. Furthermore, without the routine ability to study the human ventricular microcirculation *ex vivo*, it is a key to improve *in vivo* imaging technology to screen and characterize patients at risk and establishing medicinal strategies to ameliorate these.
