# Supplementary material for: Human coronary microvascular contractile dysfunction associates with viable synthetic smooth muscle cells
Source: Cardiovasc Res. 2021 Jun 26;118(8):1978–92. doi: 10.1093/cvr/cvab218 (PMC9239576; doi:10.1093/cvr/cvab218)
Supplement: cvab218_Supplementary_Data [file cvab218_supplementary_data.zip › suppl_data/Dora_Supplementary Materials_R3_unmarked.docx]

Human coronary microvascular contractile dysfunction associates with viable synthetic smooth muscle cells

Kim A Dora^1,^*, Lyudmyla Borysova^1^, Xi Ye^1^, Chloe Powell^1^, Timea Z Beleznai^1^, Christopher P Stanley^1^, Vito D Bruno^2^, Tobias Starborg^3^, Errin Johnson^4^, Anna Pielach^4^, Michael Taggart^5^, Nicola Smart^6^, Raimondo Ascione^2,^*

**Supplementary material online**

**Supplementary material online**

**Materials and Methods**

*Human study design*

*(1) Right atrial appendage surgical study participants.* From March 2013 to December 2017 we enrolled 88 patients (aged ≥25 and ≤80 years) with no typical obstructive coronary disease and with valvular disease requiring either aortic valve replacement (AVR) or mitral valve repair/replacement (MVR) or both (AVR+MVR) undergoing elective or urgent cardiac surgery. Exclusion criteria are outlined in **Table 1**. *Recruitment.* Right atrial appendage (RA) biopsies along with baseline blood samples were collected and anonymized. Baseline patient health information collected included patient’s age, gender, risk factors, medications, type of valve disease, and symptom status. Cardiovascular risk factors included: hypertension (need for anti-hypertension drugs), hypercholesterolaemia (need for statins), diabetes mellitus (need for oral medications or insulin), and smoking history (current or ex-smoking). Symptom status included New York Heart Association (NYHA) functional class and Canadian Cardiovascular Society (CCS) angina class. *Consenting and confidentiality.* Informed consent was obtained under and by the protocol No. 07Q160738 and No. 11SC0140. Any data information passed on to the Oxford Laboratory was anonymized. Any lab assay was carried out under blind conditions with respect to patient risk profile. The study fully complied with the Data Protection Act. *Study Settings.* The study was set up in Oxford and extended to Bristol where all the patients were recruited at the Bristol Heart Institute (BHI). The University Hospital Bristol NHS Foundation Trust sponsored the study in Bristol (Sponsor Lock code: 48676/124568/1/50). The trial was initially approved in 2012 by the Oxford Research Ethics Committee and extended to the Bristol site (Trust study No: CS/2012/4200; REC Reference No: 10/H0606/36) and amended in 2015. RA biopsy samples from recruited patients were transported to Oxford under a material transfer agreement under strict packaging, temperature regulation (approx. 10ºC) and time-limit conditions. This research complies with the Helsinki Declaration.

*Surgical methods.* Operations were carried out following standard protocols for the BHI. Baseline, surgical and anaesthetic management was according to standardised protocols.^1^ Briefly, after premedication with temazepam, anaesthesia was induced with a combination of propofol and remifentanil; muscle relaxation was achieved using vecuronium. Anaesthesia was maintained by infusion of propofol and remifentanil (5 mg remifentanil to 1 g propofol). After intubation, all participants were ventilated mechanically using routine volume control settings. Median sternotomy was then undertaken as per routine. Anticoagulation was achieved via IV heparin bolus to achieve an activated clotting time >400 s. Right atrial biopsy (approx. 1 cm^2^) was always collected before starting cardiopulmonary bypass by positioning first a purse-string suture at the tip of the right atrial appendix, followed by biopsy collection and prompt storage within 5-10 s in the preservation solution on ice and then couriered from Bristol to Oxford where they were assessed within 2-4 hours following collection. The surgical site was then used for cardiopulmonary bypass (CPB) cannulation (AVR) or tied off and over sewn (MVR) (**Figure 1**). All the subsequent aspects of surgery and postoperative management of the patients were according to standardised protocols did not affect the outcome of this study.

*(2) Left ventricle biopsies from organ donors.* Human heart tissue (Newcastle) was provided by The Newcastle Institute of Transplantation Tissue Biobank, 17/NE/0022, Research Ethics Committee North East-Newcastle and North Tyneside 1 Research Ethics Committee. *Patient details*. Females aged 56, 63 and 71 yrs. Two died of intracranial haemorrhage and one of respiratory failure, and two had a history of hypertension. Hearts were flushed with cold St Thomas No 2 Cardioplegic solution (in mmol/L): 110.0 NaCl, 10.0 NaHCO_3_, 16.0 KCl, 16.0 MgCl_2_, 1.2 CaCl_2_ (pH 7.8) and transported on ice. Left ventricular biopsies (>1cm^2^) were collected from the endocardial surface, and together with RA biopsies were immediately placed in preservation solution on ice, couriered from Newcastle to Oxford, and assessed within 5-8 hours following collection. Once the biopsy was removed, the protocols matched the strict procedures used for the human cardiac surgery RA biopsies, except the courier time was longer.

*Porcine study design*

All the animal procedures were undertaken at the University of Bristol large animal facilities from 2013-2018, including the Translational Biomedical Research Centre (TBRC), a preclinical large animal facility operating at Home Office, Good Laboratory Practice and clinical healthcare standards (http://www.bristol.ac.uk/health-sciences/research/tbrc/). All procedures were approved by University of Bristol Research Ethics committee and performed in accordance with the Guide for the Care and Use of Laboratory Animals,^2^ the United Kingdom Animal (Scientific Procedures) Act, 1986, under Home Office project licences PPL 30/2854, PPL 30/3064, and PPL 7008975, , and conform to the guidelines from Directive 2010/63/EU of the European Parliament on the protection of animals used for scientific purposes. Procedural aspects were as previously reported.^3^ Briefly, juvenile, 5-6 month old female Landrace White pigs (weight range 45–75 kg) were subject to general anaesthesia (pre-medication with ketamine, i.m. 15–20 mg/kg, induction with propofol i.v. 16–20 mg/kg and maintained with isoflurane in oxygen with the vaporizer set at 2%).

*(1) Atrial appendage study*. Animals recruited in the study were undergoing cardiac surgery with CPB procedures (PPL 30/2854) from which right atrial biopsies were collected following median sternotomy and IV heparin (target INR >400 s) prior to CPB.

*(2) Left ventricle study*. Animals recruited in the study were from within a control group of pigs undergoing cardiac surgery with CPB procedures (PPL 30/3064 and PPL 7008975) from which left ventricular biopsies from the endocardial surface could be rapidly collected at termination.

All biopsies were at least 1cm^2^. At the completion of each surgical protocol, and while still under general anaesthesia and full monitoring, animals underwent median sternotomy to expose the heart, the aorta and both superior and inferior vena cava. Euthanasia involved cardioplegic arrest using cold (0-4˚C) crystalloid solution in the aortic root after aortic clamping and concomitant exsanguination via the inferior vena cava. All porcine biopsies were collected, packed and promptly couriered to Oxford using the same approach used for human RA and LV biopsies.

*Human and porcine sample collection, transfer and dissection*

Human and porcine samples (approx. 1 cm^2^) were all collected at clinical standards during surgery and immediately placed in ice-cold nominally Ca^2+^-free HEPES-buffered solution containing (in mmol/L): 130 NaCl, 5 KCl, 1.2 MgCl_2_, 10 HEPES, 10 glucose, pH adjusted to 7.40 ± 0.02 with NaOH (at 37ºC), and transported to the laboratory in Oxford at ~10 ºC, rigorously maintained at this temperature until use (usually within 2-4 h from Bristol, within ~8 h for organ donor biopsies from Newcastle). Once in the laboratory, each human or porcine sample was transferred to a chilled dissecting dish (10ºC) containing MOPS buffer (in mmol/L): 145.0 NaCl, 4.7 KCl, 2.0 CaCl_2_, 1.17 MgSO_4_, 5.0 MOPS, 1.2 NaH_2_PO_4_, 5.0 glucose, 2.0 pyruvate, 0.02 EDTA, 2.75 NaOH (pH 7.40 ± 0.02 at 37°C). The atrial appendage or ventricle was opened and pinned for dissection of IMCAs from within trabeculae (pectinate or papillary muscle) or the subendocardial tissue, respectively, under magnification. Arteries were carefully cleared of surrounding tissue, avoiding side branches, and transferred to a myograph chamber (2 mL, RC-27 Warner Instruments, Hamden, CT) containing chilled MOPS buffer, held within the stage of an Olympus microscope, as previously described using other tissue.^4^ For all experiments, IMCAs were cannulated onto glass pipettes (outer diameter up to 100 µm), and tied with 11/0 sutures (Ethicon). The lumen was gently flushed to clear any blood, prior to cannulating the distal end. The temperature was slowly raised to 36.6 ± 0.3°C, before pressurizing arteries to 80 mmHg using a gravity-fed pressure tower. The IMCA was longitudinally stretched (final length usually 800-1200 µm), and left to equilibrate for 60 min. During this period, each IMCA was checked for leaks by either closing the pressure-head and monitoring wall deflation for 1 min and by measuring the volume flux through the arterial wall over 60 min, established as the decrease in volume held at the pressure-head.^5^ Only IMCAs with minimal transmural leaks (<5% deflation in 1 min, or <5 µL/h) were used further. All diameter and leak measurements were made at 36.5-37.0ºC. As such, all IMCAs in the study were first assessed for myogenic tone, before assessment of EC function (vasodilation to bradykinin) and/or SMC constriction (to KCl). Whilst still pressurized, each artery was then processed for either live/dead stains, EC/SMC Ca^2+^, immunohistochemistry (IHC, phalloidin or phenotype) and/or electron microscopy (EM) as shown in **Figure 1**.

*Confocal microscopy studies*

In all studies, IMCAs were visualized using Olympus linescan confocal microscopes (FV300, FV500, FV1000 or FV1200). When studying function, arteries were imaged with transmitted light using a 10x Olympus (0.40 NA, 3.1 mm WD) objective and digital zoom (usually 1.5x) and recorded using Fluoview software (Olympus, Tokyo, Japan) at 1 Hz. The structure of IMCAs was imaged using a 40x (1.15 NA, 0.25 mm WD) water immersion Olympus objective, obtaining z-stacks through the arterial wall in 0.3 to 0.5 µm steps. Fluorescent dyes were used to identify live cells (either calcein or fluo-8, excitation 488 nm), and live and/or dead cell nuclei (Hoechst 33342, excitation 405 nm; and/or propidium iodide, PI, excitation 543 nm). Post fixation, F-actin was labelled with phalloidin (excitation 543 nm), nuclei with Hoechst 33342, and elastin with Alexa Fluor^®^ 633 hydrazide (AF-633, excitation 633 nm), as previously.^6^

*SMC Test 1: Functional vascular reactivity studies*

The hydraulic conductivity of IMCAs was calculated from the volume of fluid leaving the arterial lumen over 1-5 h at 80 mmHg luminal pressure together with the arterial length and inner diameter, as previously.^5^ Myogenic tone (MT) was assessed as the tone developed in response to 80 mmHg luminal pressure, relative to the maximum diameter of arteries. The outcome of this test established how the experiment then progressed, according to the flow chart depicted in **Figure 1**. Only those IMCAs with ≥10% MT were considered to pass this first test, and were used for assessment of endothelium-dependent dilation to bradykinin (0.1 fmol/L to 1 µmol/L). Smooth muscle cell (SMC) function was further assessed in a subgroup of arteries, which either passed or failed SMC Test 1, as the vasoconstriction to 45 mmol/L isotonic KCl (added to the superfusion, NaCl substituted by KCl). When observing constriction and dilation of h-RA-IMCAs it was clear that the SMCs were changing length in unusual patterns. While some artery _walls_ only moved away from the centre in a radial manner, others seemed to move sideways without changing diameter, and others seemed to have combined responses, ending up with a twisting motion. The striking contractile behaviour of h-RA-IMCAs was depicted by the direction of constriction, whether it was radial (circumferential, to reduce diameter), longitudinal (with no clear change in diameter but clear inward usually twisting movement) or both together to give a “concertina-type” twisting motion. The longitudinal movement could also be observed as lengthening during dilation, often before the onset of radial dilation. Hence both the development and reversal of MT were assessed to categorize arteries.

*Analysis.* The 10x objective and digital zoom of 1.5 produced 1024 x 300 pixel images at 1 Hz. At this setting, each pixel was 1.12 µm^2^, the limit of movement resolution. IMCA radial and/or longitudinal motion was measured offline using Imaris software (version 8.0.2, Bitplane) using the measure line feature. *Analysis for radial movement*. The constriction response to luminal pressure or KCl are expressed as the percentage MT ((100 – developed tone) / maximum diameter x 100) or percentage vasoconstrictor tone ((100 – vasoconstrictor tone) / maximum diameter x100). The dilation evoked by bradykinin is expressed as a reversal of MT, with 100% dilation equivalent to the maximum inner diameter of each IMCA ((dilator tone – MT) / (maximum diameter – MT) x 100). Values are the mean ± SEM of n patient or pig samples, one artery per sample. *Analysis for longitudinal movement*. Clearly, as the arteries were cannulated onto two pipettes which were held in a stable position, and a force transducer was not attached to the pipettes, we could not provide clear measurements of this contraction. However, by careful frame-by-frame analysis we could see the longitudinal movement of the wall. This tended to occur in small sections of the artery, not uniform along the full length of the artery. The saved files were played forwards and backwards until three positions along the length could be visually tracked over time. By placing the first marker in a defined position on the artery wall, the sequence could be played until motion was observed, or not, and the second marker then placed, along the horizontal axis only. A grid could be displayed on the images, which aided this measurement, especially if radial constriction also occurred. The distance was calibrated, and values recorded. If more than one region moved, the average was taken, zero values not included. If no longitudinal motion was observed a value of zero was recorded. This was performed for all the arteries that developed either radial or longitudinal MT, and if tone developed, the accompanying response to bradykinin was also analyzed for comparison. Individual values were plotted, which identified a clear 5 µm cutoff point to separate radial (R) only vs radial plus longitudinal (R+L) constriction, well within our resolution limit for distance. These separated values are shown as the mean ± SEM of n patient samples, one artery per sample. Interestingly the structure of the artery wall could be seen in the transmitted images, and the longitudinal movement often associated with the presence of abnormal ‘reefs’ of cells within the lumen.

*SMC Test 2: 3D structure using confocal fluorescence microscopy*

Studies of SMC orientation were carried out following fixation of cannulated IMCAs at 80 mmHg with 2% (wt/vol) paraformaldehyde for 10 min at 36.6 ± 0.3°C, washing with phosphate-buffered saline (PBS) before further study. Whilst still cannulated, IMCAs were exposed to phalloidin and other antibodies in the incubation chamber to label from the outside of arteries, and were always also pumped into the lumen of arteries to label the ECs and inner SMC layers. IMCAs were incubated with phalloidin-TRITC (6.4 µmol/L) or antibodies overnight at 4°C. Prior to imaging, nuclei were stained with Hoechst 33342 (16 µmol/L), and elastin with Alexa Fluor^®^ 633 hydrazide (AF-633, 250 nM), and luminal pressure was reintroduced to inflate the arteries. Anti-K_Ca_3.1 (1:100 [2 µg/mL] rabbit polyclonal anti-rat K_Ca_3.1, Alomone Laboratories, APC-064, Lot # AN0402; with Alexa Fluor^®^ 488 secondary antibody, 1:100 goat anti-rabbit IgG, Invitrogen, A-11008) was used to mark ECs, as previously,^7^ and was found to label the inner-most layer of cells in at least 3 human RA-IMCAs. In separate experiments, the phenotypic state of SMCs was assessed by double- and triple-labeling markers for synthetic and contractile proteins. Combinations of primary antibodies for α-smooth muscle actin (α-SMA, 1:400 [2.5-3.8 µg/mL], mouse monoclonal-Cy3, Sigma C6198, Lot # 127M4859V); smooth muscle myosin heavy chain (SM-MHC, 1:400 [1.25 µg/mL], rabbit monoclonal anti-human Alexa Fluor^®^ 647, Abcam, ab196982, Lot # GR275457-3); caldesmon (1:200 [0.27 µg/mL], rabbit monoclonal anti-human, Abcam ab32330, Lot # GR137647-2), labels both low (l) and high (h) molecular weight caldesmon, l-caldesmon is the synthetic SMC marker; vimentin (1:500 [2 µg/mL], rabbit polyclonal anti-human, Abcam ab45939, Lot # GR311409-1); and von Willebrand Factor (vWF, 1:400 [25 µg/mL], sheep polyclonal anti-human-FITC, Abcam, ab8822, Lot # GR316897-2) were used. Arteries on pipettes were incubated overnight with primary antibody combinations, washed with PBS, then incubated with appropriate secondary antibodies (each 1:1000, goat anti-rabbit Alexa Fluor^®^ 488, A-11007; Alexa Fluor^®^ 635, A-21071; or donkey anti-rabbit Alexa Fluor^®^ 568, A10042; ThermoFisher Scientific) at RT. Arteries were washed with PBS, pressurized and image z-stacks obtained using a x40 objective (1.15 NA, Olympus). Acquired images were colour-coded using Imaris software (version 8.0.2, Bitplane).

*SMC Test 3: 3D structure using electron microscopy*

IMCAs processed for electron microscopy were fixed at the end of functional assessment for myogenic tone and vasomotor responses. Cannulated IMCAs maintained at 80 mmHg and heated to 36.6 ± 0.3°C were first rinsed in a phosphate-free buffer containing 0.1 M sodium cacodylate and 2 mmol/L CaCl_2_ (3x 5 min) and then fixed for 1 h in a buffered solution containing fixative: 1% paraformaldehyde (Electron Microscopy Services, USA), 3% glutaldehyde (TAAB Laboratories Equipment Ltd, UK), 0.1 mol/L sodium cacodylate, and 2 mmol/L CaCl_2_. After repeated washing in the rinse buffer (3x 5 min), the artery was removed from the cannulating pipettes, transferred to a vial of buffered fixative, and agitated on a Belly Dancer^®^ orbital shaker for 1 h at room temperature, before being transferred to a cold room for continued overnight fixation, with gentle agitation. Following fixation, each artery was cut into two pieces (approximately 2 mm in length) and stored at 4ºC in fixative (2.5% glutaraldehyde + 4% formaldehyde in 0.1 mol/L sodium cacodylate buffer, pH 7.2) before processing for TEM. Samples were washed with 0.1 mol/L sodium cacodylate buffer (pH 7.2) for 15 min and then embedded in 3% low melting point agarose to prevent the tissue curling. Samples were washed with buffer 2x for 15 min, then with 50 mmol/L glycine in 0.1 mol/L sodium cacodylate buffer for 25 min to quench free aldehydes, then washed twice more with cacodylate buffer for 15 min each. Samples were then stained with 1% osmium tetroxide in 0.1 mol/L sodium cacodylate buffer for 2 h at 4°C with gentle agitation and then washed with ultrapure water 4x for 15 min each. Samples were en bloc stained with 0.5% uranyl acetate (aqueous) overnight at 4°C, then washed 3x 10 min with ultrapure water. Samples were then taken through a graded ethanol series of 30%, 50%, 70%, 80%, 90% and 95% for 10 min each followed by 3x 30 min in 100% ethanol, then gradually infiltrated with TAAB low viscosity epoxy resin starting with 25% resin in ethanol for 1 h, 50% resin for 3 h, 75% resin for 2 h and 100% resin overnight. Samples were incubated in 100% resin for a further 36 h with 5 changes of resin over this time, then embedded in Beem capsules and polymerised for 48 h at 60°C. Transverse ultrathin (90 nm) sections were obtained using a Leica UC7 ultramicrotome with a diamond knife (Diatome) and transferred to formvar-coated 2x1 copper slot grids, post-stained with Reynold’s lead citrate for 5 min and imaged on a FEI Tecnai 12 TEM operated at 120 kV using a Gatan OneView CMOS camera.

A few samples were also attached to cryo pins and prepared for examination with a Gatan 3View^®^ microtome within an FEI Quanta 250 FEG scanning electron microscope, as previously.^8^ The 3View^®^ setup^9^ allows back-scattered electron images of the block surface to be collected as the sample is microtomed *in situ*, allowing serial section TEM-like images to be collected in an automated fashion. Two data sets were acquired for each sample. An overview image was taken along with a higher magnification region of interest. The overview image size was adjusted to the arterial radial cross-section field of view, ranging from 145-223 µm (2400-3500 pixels, size 58-64 nm). The higher magnification data set was always 4000x4000 pixels 10 nm pixel size (horizontal field width 40 µm). For all samples, the cut thickness was 60 nm, corresponding to the longitudinal axis of each IMCA. Raw data was converted to an MRC file stack using IMOD.^8^ In brief, the individual image intensities were floated to a common mean and standard deviation in order to remove variation in beam intensity, or detector sensitivity that can occur during long data acquisitions. Some imaging noise was removed by standard 2-dimentional Gaussian smoothing using a 3x3 kernel. Data were examined and segmented using Imaris software (version 8.0.2, Bitplane).

*SMC Test 4: Cell viability studies*

Viability of SMCs and ECs was first classified according to their ability to uptake and de-esterify the fluorescent dye calcein AM (luminally perfused, 1 µmol/L), and by comparing labelling with cell permeant (Hoechst 33342, stains live and dead cells, 16 µmol/L) and impermeant (PI, stains dead cells, 10 µmol/L) nuclear dyes.^6^ Cells were classified as live (calcein in cytoplasm; nuclei stained with Hoechst 33342 but not PI) or dead (nuclei stained with both Hoechst 33342 and PI; no calcein); live cells were expressed as a percentage of all cells in a given image field. Image z-stacks were colour-coded using Imaris software (version 8.0.2, Bitplane).

*SMC Test 5: Intracellular Ca^2+^ studies*

After establishing the vaso-reactivity of arteries (at 37ºC, SMC Test 1), changes in arterial SMCs and/or ECs intracellular Ca^2+^ content were imaged using the fluorescent Ca^2+^ indicator fluo-8. Pressurized IMCAs were intraluminally perfused with HEPES buffer containing fluo-8 AM (10 µmol/L), and 0.05% pluronic F-127 for 35-40 min at 31ºC, before washing with MOPS buffer and de-esterification for 20 min. Selective loading of ECs was achieved by reducing the exposure time to fluo-8 AM. Images were obtained using a x40 (1.15 NA, Olympus) or x60 (1.20 NA, Olympus) water immersion objective and iXon 887 EMCCD camera (Andor Technology, UK) coupled to a Nipkow spinning disk confocal head (CSU22, Yokogawa, Japan) mounted on an inverted microscope (IX70, Olympus, Japan). After excitation at 488 nm, fluorescence emission intensity at 513-563 nm from at least 10 cells at the bottom surface of arteries were acquired at between 10 and 30 Hz (cropped from the 512 x 512 pixel chip) using Andor iQ software (version 3.5, Andor Bioimaging Division, UK). All Ca^2+^ imaging experiments were performed at 31-33ºC instead of the usual 37ºC for all diameter experiments to reduce the high rate of fluo-8 dye leakage from cells at 37ºC. Agents were added directly to a static bath, except for KCl which was rapidly switched to a warmed isotonic KCl solution. Porcine IMCAs retained some myogenic tone at 31-33ºC, and further contracted to KCl and caffeine, therefore nifedipine (1 µmol/L) was present to reduce movement, which precluded the use of KCl in these arteries. Responses to both caffeine and bradykinin, to activate Ca^2+^ release via ryanodine receptors (RyRs) and inositol trisphosphate receptors (IP_3_Rs), respectively, were obtained in the same cells, and these agents were used to distinguish longitudinally-arranged SMCs (l-SMCs) from ECs, which was confirmed by the Ca^2+^ influx in response to depolarization with KCl.

*Analysis*. Up to 6 cells per field of view were analyzed and averaged to give one *n* value per artery. Only cells that responded to KCl or caffeine (for SMCs) or bradykinin (for ECs) were included in the analysis. Data were analyzed offline using MetaMorph software (version 7.7.4.0, Molecular Devices). Subcellular regions of interest (diameter ~5 µm) were positioned both within active cells and away from cells (latter for background intensity) to obtain fluorescence intensity over time. Background-subtracted raw data are expressed as relative fluorescence (F/F_0_) by dividing fluorescence intensity (F) by an average baseline fluorescence intensity F_0_. Values are summarized as the mean ± SEM, with *n* representing the number of arteries studied.

*SMC Test 6: Contractile phenotype studies*

The experiments within and across species assessing the expression of contractile and synthetic markers were kept as constant as possible. The image acquisition settings were consistent within the 3D z-stack, allowing intra-artery comparison of r-SMC and l-SMC expression of SM-MHC or α-SMA, alongside caldesmon or vimentin. These settings were then matched as closely as possible for all arteries studied. Arteries were imaged such that the x-y axis ran along the length of the artery, which allowed multiple r-SMCs to be visualized in a given field (up to ~30 r-SMCs).

*Analysis*. Multi-channel image z-stacks (contractile and synthetic markers, plus nuclei and EC label) were analyzed offline using Imaris software (version 8.0.2, Bitplane). *Analysis of percentage synthetic r-SMCs.* By visual inspection of the cytoplasm of the cells, counts of individual r-SMCs with greater relative intensity for synthetic *versus* contractile markers within the cell, were expressed as a percentage of all r-SMCs visible in the field, providing values for percentage synthetic r-SMCs. There was often clear heterogeneity in the extent of marker expression within a given cell, consistent with observations in heterogeneity of markers within single cultured human coronary artery SMCs.^10^ Therefore even if only a subcellular region had more synthetic marker, it was counted as a synthetic cell. This approach was considered the most informative and accurate index of relative marker expression within and between arteries. *Analysis of percentage caldesmon in r-SMCs vs l-SMCs*. Separately, to calculate the the percentage of cells expressing caldesmon in r-SMC and l-SMC, the average fluorescence intensity of lines drawn across either r-SMCs or l-SMCs were used, in most cases from a plane where both r-SMCs and l-SMCs were clearly visible and separated. The same lines could be used for both contractile and synthetic markers within a given image plane. This enabled both a direct comparison of the same protein between r-SMCs and l-SMCs, and an indirect comparison between contractile and synthetic proteins.

*Materials*

NaCl, KCl, and D-glucose were purchased from Fisher Scientific (Loughborough, UK); MOPS, EDTA, CaCl_2_ 2H_2_O, pyruvate, PBS sachets, Tween20, and bovine serum albumin were purchased from Sigma (Poole, UK); MgSO_4_ 7H_2_O, NaOH, and NaH_2_PO_4_ H_2_O were purchased from VWR (Leicestershire, UK); and bradykinin was purchased from Tocris (Bristol, UK). The cellular dyes Hoechst 33342 (H3570), PI (P1304MP), DAPI (D3571), and AF-633 (A30634) were purchased from ThermoFisher Scientific (Paisley, UK), phalloidin-TRITC from Sigma-Aldrich (Dorset, UK) and fluo-8 AM from AAT Bioquest (Stratech, Newmarket, UK). Paraformaldehyde was purchased from Electron Microscope Sciences (Hatfield, PA, USA).

*Statistics*

Statistical analysis was performed using GraphPad Prism software (version 8, GraphPad Software, La Jolla, USA), where *P*<0.05 was considered significant. All specimens collected were analyzed and no experimental data were excluded from the study. Formal statistical comparisons on our unpaired data first tested for Gaussian distributions (D’Agostino and Pearson omnibus normality test), which confirmed that for all comparisons nonparametric tests should be used with no assumption of equal standard deviation. Subsequently two groups were compared with two-tailed Mann-Whitney tests, and groups of 3 or more with Kruskal-Wallis tests and Dunn’s post-test. For these analyses values are the mean±SEM. To correlate MT results with synthetic markers in r-SMCs or with other variables, univariable and multivariable linear regression models were run using R (version 3.6.0, https://www.R-project.org/) and jtools (version 2.0.1, https://cran.r-project.org/package=jtools), and as indicated, adjusted for any known risk factors (Model A: sex, type of valve disease, hypertension, hypercholesterolaemia and smoking history) and medications (Model B: statins, diuretics, Ca^2+^ channel blockers, beta-blockers, aspirin, anticoagulants, angiotensin-converting-enzyme (ACE)-inhibitors). Multiple regression linear models were obtained after a forward/backward stepwise selection process with AIC as the selection criterion for the final model. Analysis for a global validation of the linear model assumptions as well as separate evaluations of skewness, kurtosis, link function and heteroscedasticity were tested using R (package, lmSupport; function, modelAssumptions) and all were found to be acceptable. Original data points are presented as scatter plots where possible, and these data were tested for Gaussian distributions (D’Agostino and Pearson omnibus normality test or Shapiro Wilk test). For ease of visualization, the mean±SEM are shown on the scatter plots. The inclusion of all arteries, including those that did not develop myogenic tone, meant the 88 myogenic tone values for h-RA-IMCA data were not normally distributed, so we report the median with lower 95% and upper 95% confidence intervals in the figure legends using the format [median,LCI,UCI]. Other data were normally distributed and, where reported, the median shown as [median].

**References**

1. Rogers CA, Capoun R, Scott LJ, Taylor J, Jain A, Angelini GD, Narayan P, Suleiman MS, Sarkar K, Ascione R. Shortening cardioplegic arrest time in patients undergoing combined coronary and valve surgery: results from a multicentre randomized controlled trial: the SCAT trial. *Eur J Cardiothorac Surg* 2017;**52**:288-296.

2. Garger JC, Barbee RW, Bielitzki JT, Donovan JC, Hendriksen FM, Kohn DF, Lipman NS, Locke PA, Quimby FW, Turner PV, Wood GA, Würbel H, Liebig J. Guide for the Care and Use of Laboratory Animals: Eighth Edition. Washington D.C., USA: National Academies Press, 2011.

3. Gadeberg HC, Bond RC, Kong CH, Chanoit GP, Ascione R, Cannell MB, James AF. Heterogeneity of t-tubules in pig hearts. *PLoS ONE* 2016;**11**:e0156862.

4. Garland CJ, Bagher P, Powell C, Ye X, Lemmey HAL, Borysova L, Dora KA. Voltage-dependent Ca^2+^ entry into smooth muscle during contraction promotes endothelium-mediated feedback vasodilation in arterioles. *Science signaling* 2017;**10**:1-14.

5. Lucotte BM, Powell C, Knutson JR, Combs CA, Malide D, Yu ZX, Knepper M, Patel KD, Pielach A, Johnson E, Borysova L, Dora KA, Balaban RS. Direct visualization of the arterial wall water permeability barrier using CARS microscopy. *Proc Natl Acad Sci U S A* 2017;**114**:4805-4810.

6. Dora KA, Stanley CP, Al Jaaly E, Fiorentino F, Ascione R, Reeves BC, Angelini GD. Isolated human pulmonary artery structure and function pre- and post-cardiopulmonary bypass surgery. *Journal of the American Heart Association* 2016;**5**:1-9.

7. Bagher P, Beleznai T, Kansui Y, Mitchell R, Garland CJ, Dora KA. Low intravascular pressure activates endothelial cell TRPV4 channels, local Ca^2+^ events, and IK_Ca_ channels, reducing arteriolar tone. *Proc Natl Acad Sci USA* 2012;**109**:18174-18179.

8. Starborg T, Kalson NS, Lu Y, Mironov A, Cootes TF, Holmes DF, Kadler KE. Using transmission electron microscopy and 3View to determine collagen fibril size and three-dimensional organization. *Nat Protoc* 2013;**8**:1433-1448.

9. Denk W, Horstmann H. Serial block-face scanning electron microscopy to reconstruct three-dimensional tissue nanostructure. *PLoS Biol* 2004;**2**:e329.

10. Beamish JA, He P, Kottke-Marchant K, Marchant RE. Molecular regulation of contractile smooth muscle cell phenotype: implications for vascular tissue engineering. *Tissue engineering Part B, Reviews* 2010;**16**:467-491.

**Figure S1**


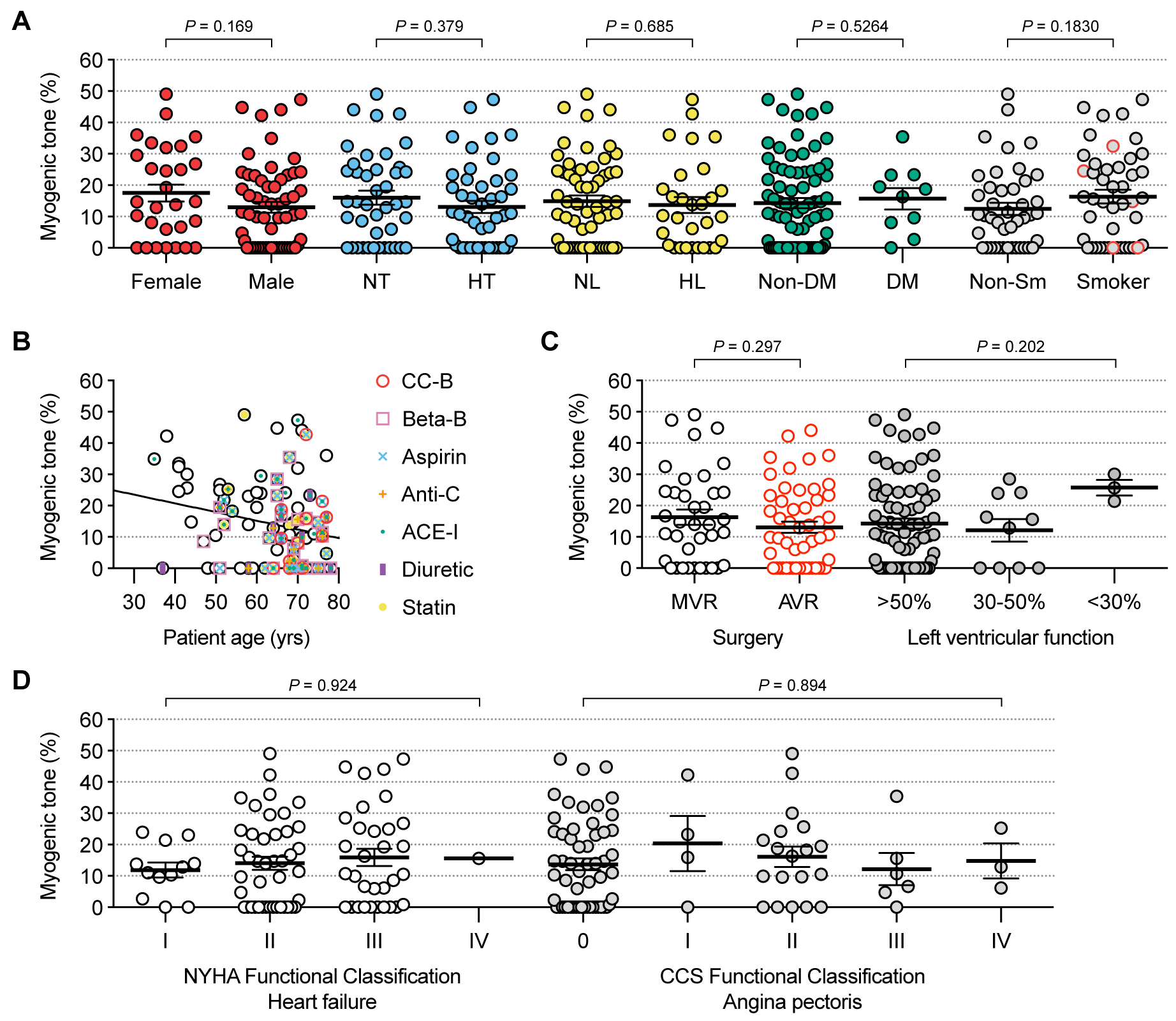


Effect of baseline risk factors on myogenic tone in h-RA-IMCAs (*n*=88). (**A**) No significant association of developed myogenic tone with either sex (female *n*=29/88), hypertension (NT: no need for treatment; Treated: on medications, *n*=45/88), hypercholesterolaemia (NL: no need for treatment; HL: on statins, *n*=31/88), diabetes (DM: treated for diabetes mellitus, *n*=10/88), or smoking (Smoker: includes smokers having ceased smoking at least 1 month prior to surgery, red circles indicate current smokers, *n*=45/88). (**B**) Detail of medications combined with plot of patient age and developed myogenic tone (*n*=88); CC-B, Ca^2+^ channel blocker (*n*=12); Beta-B, beta-blocker (*n*=25); Anti-C, anticoagulant (*n*=16); ACE-I: angiotensin converting enzyme inhibitor (*n*=29), see also **Figure 2** and **Table 1**. No significant associations of developed myogenic tone with (**C**) type of surgery (MVR: mitral valve repair/replacement; AVR: aortic valve replacement, *n*=51/88), or (**D**) patient information graded according to the New York Heart Association Functional Classification (NYHA) for heart failure and the Canadian Cardiovascular Society (CCS) for angina pectoris (see **Table 1** for *n* values). To compare means, we used a nonparametric unpaired two-tailed Mann-Whitney test for each coloured-pair or Kruskal-Wallis test with Dunn’s post-test for multiple comparisons. *P*<0.05 was considered significant.

**Figure S2**


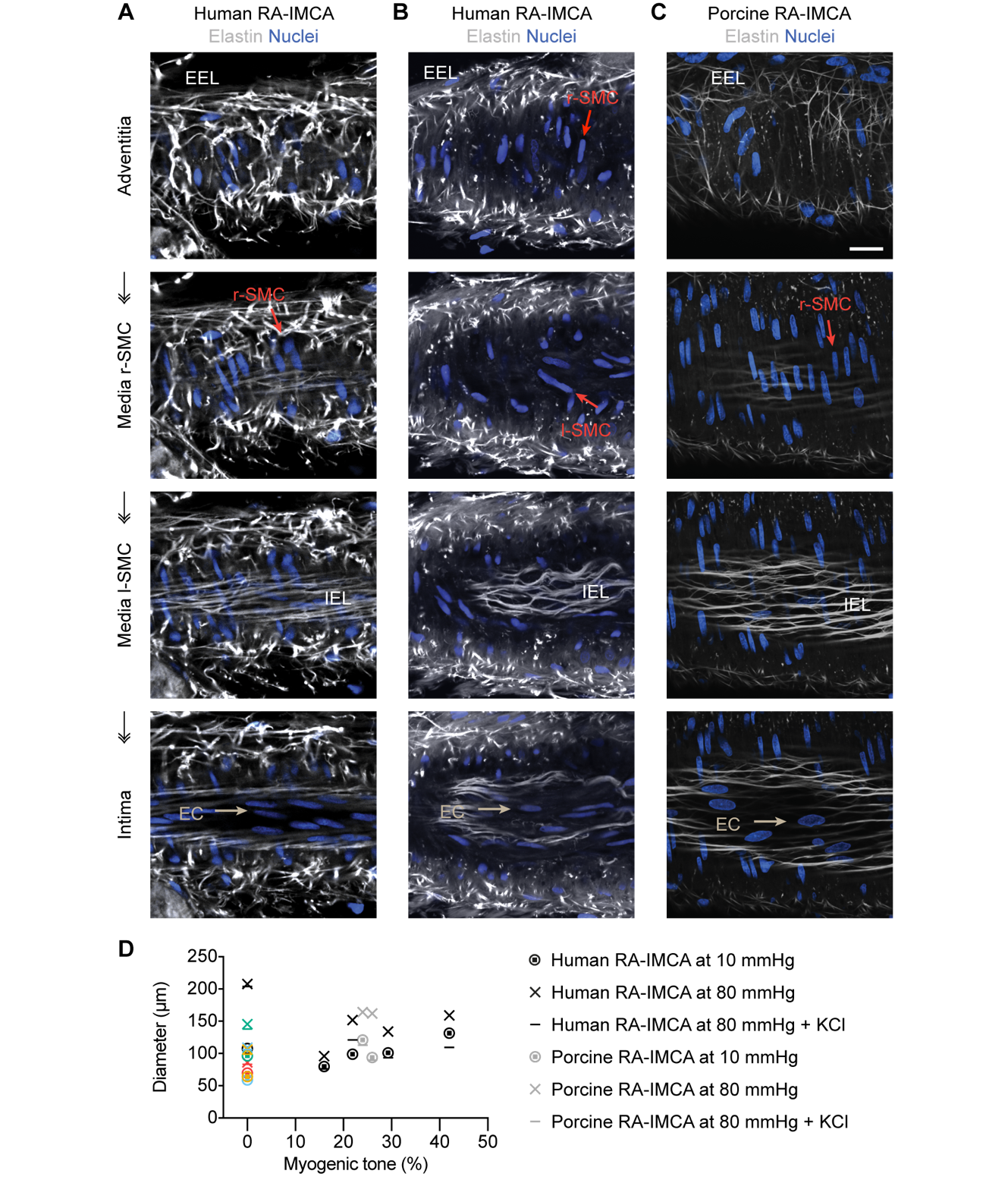


The elastic lamina separates ECs and l-SMCs (and r-SMCs) and is denser in h-RA-IMCAs compared to p-RA-IMCAs. (**A** to **C**) Confocal micrographs from isolated, cannulated and pressurized RA-IMCAs. (**A** and **B**) Partially rendered 3D images from two h-RA-IMCAs, and for comparison (**C**) a p-RA-IMCA (corresponding full 3D image, **Supplementary material online, Movie S1**). A representative micrograph is shown as the focal plane passes through the arterial wall, commencing in the adventitia, at the external elastic lamina (EEL), passing through the media to the internal elastic lamina (IEL), and finally the intimal endothelial cells (ECs) and the arterial lumen. Representative of ≥10 arteries from human and porcine atrial appendage samples. ↠, Confocal z-stack through the wall of an IMCA. (**D**) Pressure-diameter values for human (black and coloured symbols, *n*=9) and porcine (grey symbols, *n*=2) RA-IMCAs. Values for inner diameters at 10 mmHg and 80 mmHg luminally applied pressure, along with the diameter during isotonic 45 mmol/L KCl (KCl) are plotted against the developed myogenic tone in each artery. Colours represent RA-IMCAs from different patients (all no MT). The ability to develop myogenic tone was not related to the magnitude of passive wall distension.

**Figure S3**

**
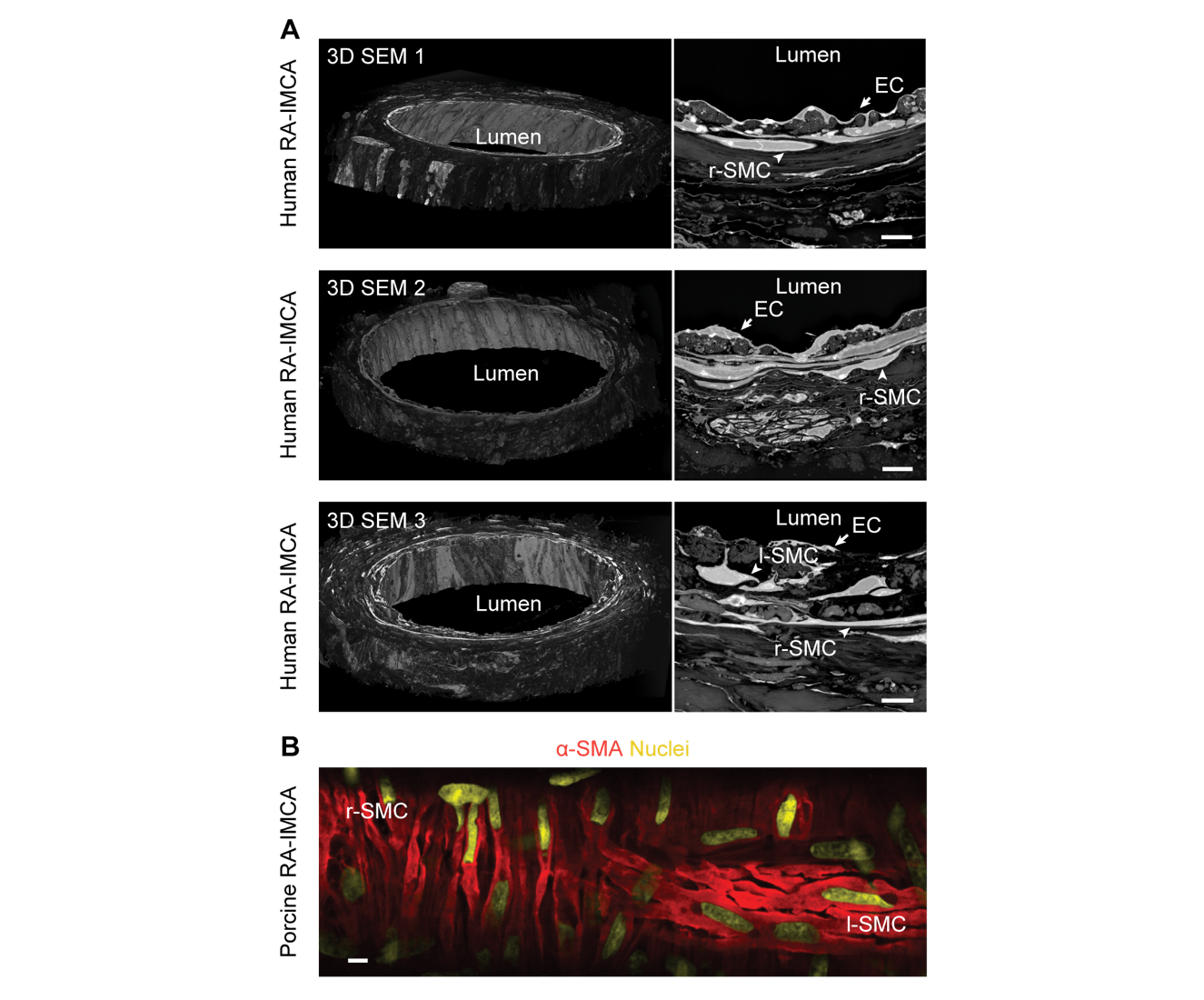
**

3D IMCA structure reveals l-SMCs. (**A**) Human RAA-IMCAs shown in **Figure 4** (SEM 1 to 3) imaged by SBF-SEM, see **Supplementary material online, Movies S2 to S5**. The measurements of inner diameter of pressurized arteries live, before fixation and then once processed and imaged using the SBF-SEM are SEM 1: 116 µm and 120 µm; SEM 2: 133 µm and 137 µm; SEM 3: 186 µm and 192 µm. Note that the position along the artery length could not be precisely matched between measurements. (**B**) Isolated cannulated porcine RA-IMCA labelled for α-SMA and nuclei. r-SMCs overlap l-SMC in the center of the image. Bar=5 µm in all images.

**Figure S4**

**
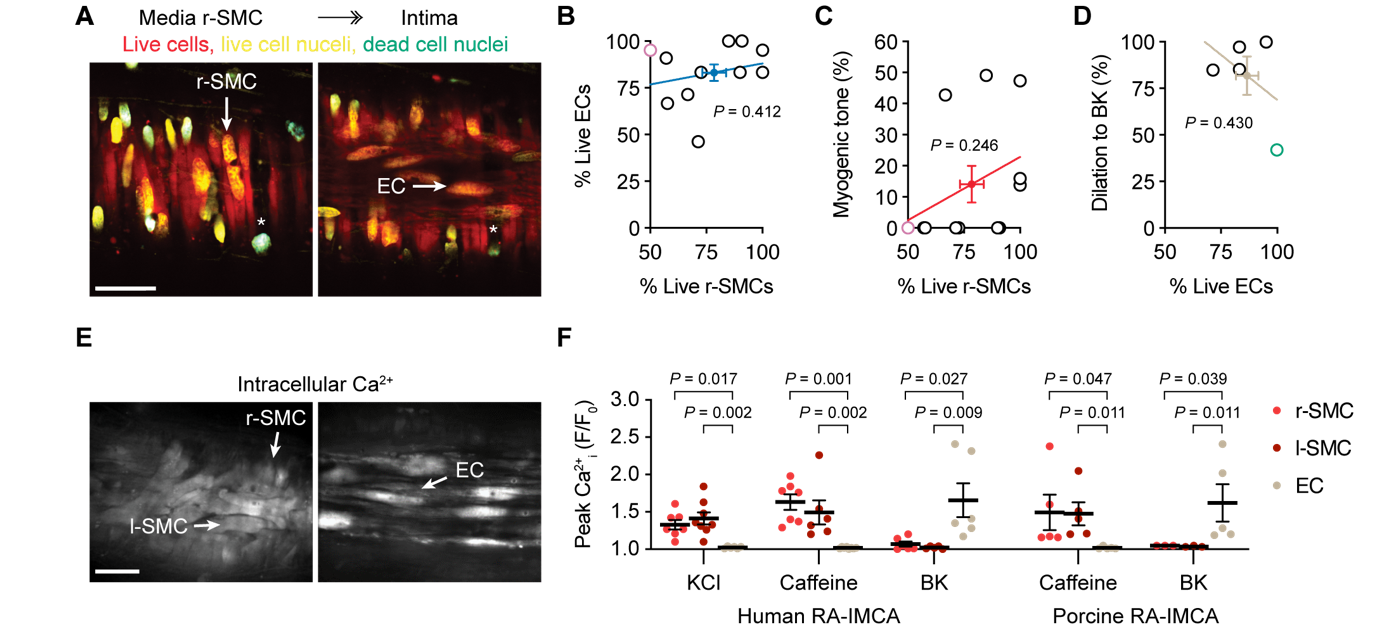
**

Vaso-inactivity of h-RA-IMCAs is not explained by poor r-SMC viability or an inability to elevate cytosolic Ca^2+^. (**A**) Confocal micrographs from a cannulated and pressurized human RA-IMCA incubated with Hoechst 33342 to label both live and dead cell nuclei, propidium iodide (PI) for dead cell nuclei and calcein for live cells. The asterisk indicates a non-viable r-SMC; this artery (**A**) is indicated by the pink symbols in **B** and **C**; bar=20 µm. **B-D**, Summary of h-RA-IMCAs showing the relationship between the percentage of live r-SMCs, and the percentage of live ECs (**B**), and the percentage MT (*n*=12, **C**). (**D**) Summary of the response to 10 nM BK assessed from developed MT (*n*=5); the h-RA-IMCA indicated by the green symbol fully dilated to 100 nM BK. (**E**) Fluorescence micrographs of cannulated and pressurized h-RA-IMCAs loaded with fluo-8 to reveal r-SMCs and l-SMCs (left panel) and ECs (right panel, different artery); bar=20 µm. (**F**) Summary of intracellular Ca^2+^ responses to agonists in h-RA-IMCAs (5.6±2.4% MT, *n*=12; for three cell types: KCl, *n*=6-8, MT 5.5±3.0%, *n*=9; caffeine, *n*=6-8, MT 5.4±3.0%, *n*=9; BK, *n*=5-6, MT 8.0±3.6%, *n*=7) and p-RA-IMCAs (with MT, *n*=4-6) RA-IMCAs. KCl, isotonic, 45 mmol/L; caffeine, 1 mmol/L; BK, 100 nmol/L (h-RA-IMCA) or 1 nmol/L (p-RA-IMCA). Linear regression in **B**-**C**; nonparametric Kruskal-Wallis test with Dunn’s post-test in **F**. ↠, Confocal z-stack through the wall of an IMCA.

**Table S1**

**Linear Regression Model: factors affecting myogenic tone in h-RA-IMCAs (*n*=88)**

|  | Estimates | SEM | p-value |  | Estimates | SEM | p-value |
| --- | --- | --- | --- | --- | --- | --- | --- |
|  |  | | |  |  | | |
| **Risk Factors** | **Univariable** | | |  | **Multivariable Model A^†^** | | |
|  |  |  |  |  |  |  |  |
| **Age** | -0.28 | 0.13 | **0.04** |  | -0.27 | 0.13 | **0.04** |
| Female Sex | 4.53 | 3.09 | 0.15 |  | 5.27 | 3.07 | 0.09 |
| MV disease | 0.65 | 0.44 | 0.14 |  |  |  |  |
| Hypertension | -2.98 | 2.93 | 0.31 |  |  |  |  |
| Hypercholesterolaemia | -1.23 | 3.11 | 0.69 |  |  |  |  |
| Diabetes Mellitus | 1.38 | 4.64 | 0.77 |  |  |  |  |
| Active Smoker | -0.10 | 6.37 | 0.98 |  |  |  |  |
| Previous Smoker | 3.94 | 2.93 | 0.18 |  | 5.03 | 2.90 | 0.09 |
|  |  |  |  |  |  |  |  |
| **Medications** | **Univariable** | | |  | **Multivariable Model B^#^** | | |
|  |  |  |  |  |  |  |  |
| **Age** | -0.28 | 0.13 | **0.04** |  | -0.20 | 0.14 | 0.16 |
| Statins | -3.25 | 3.02 | 0.29 |  |  |  |  |
| Diuretics | -7.44 | 3.36 | **0.03** |  | -7.37 | 3.83 | 0.06 |
| Ca^2+^ channel blocker | -2.71 | 4.28 | 0.52 |  |  |  |  |
| Beta-blocker | -6.72 | 3.19 | **0.04** |  | -4.56 | 3.28 | 0.17 |
| Aspirin | -2.21 | 3.06 | 0.53 |  |  |  |  |
| Anticoagulants | -6.08 | 3.76 | 0.11 |  |  |  |  |
| ACE-inhibitors | 0.09 | 3.13 | 0.98 |  | 4.75 | 3.38 | 0.16 |
|  |  |  |  |  |  |  |  |

^†^, Multivariable Model A: adjusted for all known risk factors followed by stepwise backward/forward selection; ^#^, Multivariable Model B: adjusted for all medications followed by stepwise backward/forward selection; ACE, angiotensin converting enzyme.

**Movie S1.** Confocal z-stack of the 3D arrangement of elastin in an isolated, cannulated and pressurized porcine IMCA.

**Movie S2**. SBF-SEM z-stack of the 3D arrangement of the wall of an isolated, cannulated and pressurized human IMCA. Corresponds to Patient 4 in **Figure 4**, low resolution, inner diameter 120 µm.

**Movie S3.** SBF-SEM z-stack of the 3D arrangement of the wall of an isolated, cannulated and pressurized human IMCA. Corresponds to Patient 4 in **Figure 4**, high resolution.

**Movie S4**. SBF-SEM z-stack of the 3D arrangement of the wall of an isolated, cannulated and pressurized human IMCA. Corresponds to Patient 6 in **Figure 4**, high resolution, inner diameter 140 µm.

**Movie S5**. SBF-SEM z-stack of the 3D arrangement of the wall of an isolated, cannulated and pressurized human IMCA. Corresponds to Patient 7 in **Figure 4**, high resolution, inner diameter 190 µm.

**Movie S6**. Confocal z-stack of the 3D arrangement of cells within an isolated, cannulated and pressurized human IMCA. Red corresponds to α-SMA, blue to caldesmon, yellow nuclei (**Figure 5**). Full x-axis 160 µm.

**Movie S7**. Confocal z-stack of the 3D arrangement of cells within an isolated, cannulated and pressurized porcine IMCA. Red corresponds to α-SMA, blue to caldesmon, yellow nuclei (**Figure 5**). Full x-axis 160 µm.
